# Metabolic score for insulin resistance and the incidence of cardiovascular disease: a meta-analysis of cohort studies

**DOI:** 10.3389/fendo.2025.1699985

**Published:** 2025-10-17

**Authors:** Ye He, Jiading He, Dongping Chen, Jianmin Xiao

**Affiliations:** ^1^ Department of Cardiology, The Dongguan Affiliated Hospital of Jinan University, Binhaiwan Central Hospital of Dongguan, Dongguan, Guangdong, China; ^2^ Department of Cardiology, The First Affiliated Hospital of Jinan University, Guangzhou, Guangdong, China; ^3^ Central Laboratory, The Dongguan Affiliated Hospital of Jinan University, Binhaiwan Central Hospital of Dongguan, Dongguan, Guangdong, China

**Keywords:** METS-IR, insulin resistance, cardiovascular disease, coronary artery disease, stroke, meta-analysis, cohort studies, dose-response relationship

## Abstract

Cardiovascular disease (CVD) remains the leading global cause of mortality, with insulin resistance as a pivotal metabolic risk factor that promotes endothelial dysfunction, inflammation, and atherosclerosis via mechanisms such as impaired nitric oxide signaling and enhanced oxidative stress. The metabolic score for insulin resistance (METS-IR), a non-insulin-based index derived from fasting blood glucose, triglycerides, high-density lipoprotein cholesterol, and body mass index, offers a practical surrogate for assessing insulin sensitivity. However, its association with incident CVD has not been systematically evaluated in a meta-analysis. This meta-analysis aimed to quantify the relationship between baseline METS-IR and the incidence of composite CVD, coronary artery disease (CAD), and stroke in adults without baseline CVD, including categorical, continuous, and dose-response analyses. We searched PubMed, EMBASE, Cochrane Library, and Web of Science up to August 2, 2025, for cohort studies. Hazard ratios (HRs) were pooled using random-effects models to account for heterogeneity for highest versus lowest METS-IR categories and per standard deviation (SD) increment. Nonlinear dose-response relationships were modeled with restricted cubic splines. Heterogeneity, sensitivity, and publication bias were assessed. Eight cohort studies involving 437,283 participants were included. Highest vs. lowest METS-IR was associated with increased risks (HR [95% CI]; I²): composite CVD (1.65 [1.36-2.02]; 85.6%), CAD (1.82 [1.50-2.20]; 59.7%), stroke (1.47 [1.19-1.83]; 76.3%). Per SD increment: composite CVD (1.16 [1.10-1.22]; 70.7%), CAD (1.18 [1.11-1.25]; 52.4%), stroke (1.13 [1.06-1.19]; 67.9%). Dose-response analyses revealed a nonlinear association for CAD (P for nonlinearity: 0.011), marginal nonlinearity for stroke (P: 0.072), and suggested nonlinearity for composite CVD (P: 0.145), with inflection points at METS-IR values of 40.56 (composite CVD), 38.24 (CAD), and 48.88 (stroke), beyond which risks appeared to accelerate. Elevated METS-IR independently predicts higher incidence of composite CVD, CAD, and stroke with nonlinear thresholds for CAD, marginal nonlinear thresholds for stroke, and potential nonlinear thresholds for composite CVD, despite moderate-to-high heterogeneity, supporting its integration into risk stratification and preventive strategies for metabolic health management.

**Systematic review registration:**

https://www.crd.york.ac.uk/PROSPERO/display_record.php?ID=CRD420251104293, identifier CRD420251104293.

## Introduction

Cardiovascular disease (CVD) is the leading cause of death globally, accounting for approximately 17.9 million deaths annually, with projections estimating an increase to over 23 million by 2030 (1). As a core component of metabolic syndrome and type 2 diabetes, insulin resistance is considered a primary driver of CVD (2–4). It contributes to endothelial dysfunction, inflammation, and accelerated atherosclerosis through mechanisms such as impaired nitric oxide signaling and enhanced oxidative stress (2–4).

Traditional methods for assessing insulin resistance, such as the Homeostatic Model Assessment for Insulin Resistance (HOMA-IR), rely on measurements of fasting insulin levels (5). However, fasting insulin is not routinely measured in clinical practice. To address this limitation, several novel insulin resistance surrogate indices that do not require insulin measurement have been developed, including the triglyceride-glucose (TyG) index, triglyceride-glucose-body mass index (TyG-BMI) index, and metabolic score for insulin resistance (METS-IR) (5–7). Numerous meta-analyses synthesizing evidence on indices such as TyG and HOMA-IR have demonstrated consistent associations with CVD risk (8–10). Nonetheless, it remains unclear which non-insulin-dependent surrogate index exhibits superior predictive ability for CVD. Moreover, no meta-analysis has yet summarized the association between METS-IR and CVD incidence risk. Individual cohort studies have reported varying effect sizes for METS-IR in predicting composite CVD, coronary artery disease (CAD), and stroke (11, 12). However, these studies are often limited by small sample sizes, geographic specificity, or inadequate adjustment for confounding factors such as hypertension and lipid-modifying treatments (11, 12). Compared with other non-insulin-dependent surrogate indices such as TyG, METS-IR has shown superior predictive value for visceral obesity, incident diabetes, and metabolic disorders (7). This advantage has been validated against the gold standard for insulin resistance assessment—the hyperinsulinemic-euglycemic clamp technique (7). In terms of applicability, METS-IR can be manually calculated using a straightforward formula based on routinely available clinical measurements (fasting blood glucose (FBG), triglycerides (TG), high-density lipoprotein cholesterol (HDL-C), and BMI), making it practical for everyday clinical use without specialized equipment or analyses, as demonstrated in validation studies across diverse populations (7, 13–15). In diverse populations, higher METS-IR levels are associated with increased arterial stiffness and subclinical atherosclerosis (13–15). Therefore, a rigorous meta-analysis is warranted to quantify the predictive role of METS-IR in CVD incidence risk.

This meta-analysis aims to evaluate the association between baseline METS-IR and the incidence of CVD outcomes (including composite CVD, CAD, and stroke) in adult populations without baseline CVD, as well as to explore the dose-response relationship between this index and those outcomes. Ultimately, these findings will provide evidence-based insights to facilitate the integration of METS-IR into global frameworks for CVD prevention.

## Methods

The protocol was registered with PROSPERO (International Prospective Register of Systematic Reviews, https://www.crd.york.ac.uk/PROSPERO) under registration number CRD420251104293. This meta-analysis was conducted following Preferred Reporting Item for Systematic Review and Meta-Analysis 2020 guidelines (PRISMA 2020). (**Supplementary Material: Table S1**).

### Literature search

Articles published from the inception of the databases up to August 2, 2025, were retrieved from PubMed, EMBASE, The Cochrane Library, and Web of Science using the following title terms: “cardiovascular disease”, “CVD”, “coronary artery disease”, “Coronary Disease”, “CAD”, “CHD”, “stroke”, “Ischemic Attack, Transient”, “Peripheral Arterial Disease”, “METS-IR”, and “Metabolic Score for Insulin Resistance”. The search was conducted by combining MeSH terms and free-text words, with no language restrictions applied. The detailed search strategy is described in **Supplementary Material: Table S2**.

### Study selection

This systematic review process followed a two-stage screening approach in line with PRISMA guidelines to ensure comprehensive and reproducible study selection. Two researchers independently conducted the entire process from literature search and selection to data analysis. We used Zotero 7.1-beta.41 + 355c61e6d (64-bit) software (Corporation for Digital Scholarship, Vienna, Virginia, USA) to organize all studies. After automatically and manually removing duplicates, relevant literature was initially screened by examining titles and abstracts. Subsequently, full texts of the preliminarily screened literature were reviewed to determine the final eligible studies. Any discrepancies during this process were resolved by a third reviewer. The inclusion criteria for studies were as follows: (1) being a cohort study published as a full text; (2) including an adult population without CVD at baseline; (3) measuring METS-IR at baseline and reporting specific values; (4) having the primary outcome as a composite outcome of CVD and secondary outcomes as individual CVD events; and (5) reporting hazard ratios (HRs) after adjusting for potential confounding factors. The formula for calculating METS-IR is: ln [(2×FBG (mg/dL)) + TG (mg/dL)] × BMI (kg/m²))/(ln [HDL-C (mg/dL)]) (7). The composite outcome of CVD was defined as the incidence of CAD, stroke, transient ischemic attack, and peripheral arterial disease. The diagnosis of CAD, stroke, transient ischemic attack, and peripheral arterial disease was consistent with the criteria of the original studies. Studies were excluded if they were reviews, meta-analyses, abstract-only articles, or focused on other outcomes. If there was an overlap in the population between different studies from the same registry or group, only the study with the largest sample size was included.

### Data extraction and quality assessment

Two authors independently extracted relevant information from eligible studies, and any discrepancies were resolved by consensus. The extracted data included: (1) first author’s name, year of publication, and country; (2) characteristics of the study design; (3) participant characteristics, including health status, sample size, age, and gender ratio; (4) METS-IR analysis model; (5) follow-up duration; (6) reported outcomes and outcome validation methods; and (7) confounding factors adjusted for in multivariate analysis. For the included cohort studies, the Newcastle-Ottawa Scale (NOS) was used to assess the quality and strength of evidence for each outcome. This scale, which ranges from 1 to 9 points, evaluates the quality of cohort studies based on the selection of study groups, comparability between groups, and ascertainment of the outcome of interest (16).

### Statistical analysis

Hazard ratios (HRs) and their corresponding 95% confidence intervals (CIs) were used as the general measure to assess the association between baseline METS-IR and the incidence of CVD, CAD, stroke, transient ischemic attack, or peripheral arterial disease in the adult population. For studies analyzing METS-IR as a categorical variable, the HR for CVD incidence comparing the highest METS-IR level to the lowest was extracted. For studies analyzing METS-IR as a continuous variable, the HR for CVD incidence per 1 standard deviation (SD) increase in METS-IR was extracted. Data on HRs and their standard errors were calculated from 95% CIs or P-values; these were then log-transformed for variance stabilization and distribution standardization (17). Heterogeneity was evaluated using the I² statistic and Cochran’s Q test (18); if I² > 50% or P < 0.10, indicating significant heterogeneity, a random-effects model was used to pool HR data. Otherwise, a fixed-effects model was applied (19). Sensitivity analyses were performed by excluding one individual study at a time to test the stability of the results (20). If more than 10 studies were included for each outcome, subgroup analyses were conducted stratified by gender, age, and diabetes status (19). Publication bias was graphically assessed using funnel plots. Additionally, when necessary, Egger’s test (for ≥10 included studies) and the trim-and-fill method were applied to further evaluate publication bias (19, 21–23). The nonlinear mixed-effects model approach proposed by Jiang et al. was adopted to directly integrate data from all studies. Restricted cubic splines (RCS) were used to capture the nonlinear association between METS-IR and the risk of outcome incidence, while incorporating study-level random effects to account for between-study variation, thereby achieving overall modeling of the dose-response relationship (24). Meanwhile, second derivative analysis was used to precisely locate the inflection points of the curve (25). When METS-IR was reported in categorical intervals, for closed intervals, the midpoint of the upper and lower bounds of the interval was taken as the exposure level; for open intervals, the interval length was set to that of the adjacent group, and the midpoint was used as the mean exposure level (26). Meta-analysis and statistical analyses were performed using R software version 4.5.1 (R Core Team, Vienna, Austria). A P-value < 0.05 was considered statistically significant.

## Results

### Study selection process

**Figure 1:**
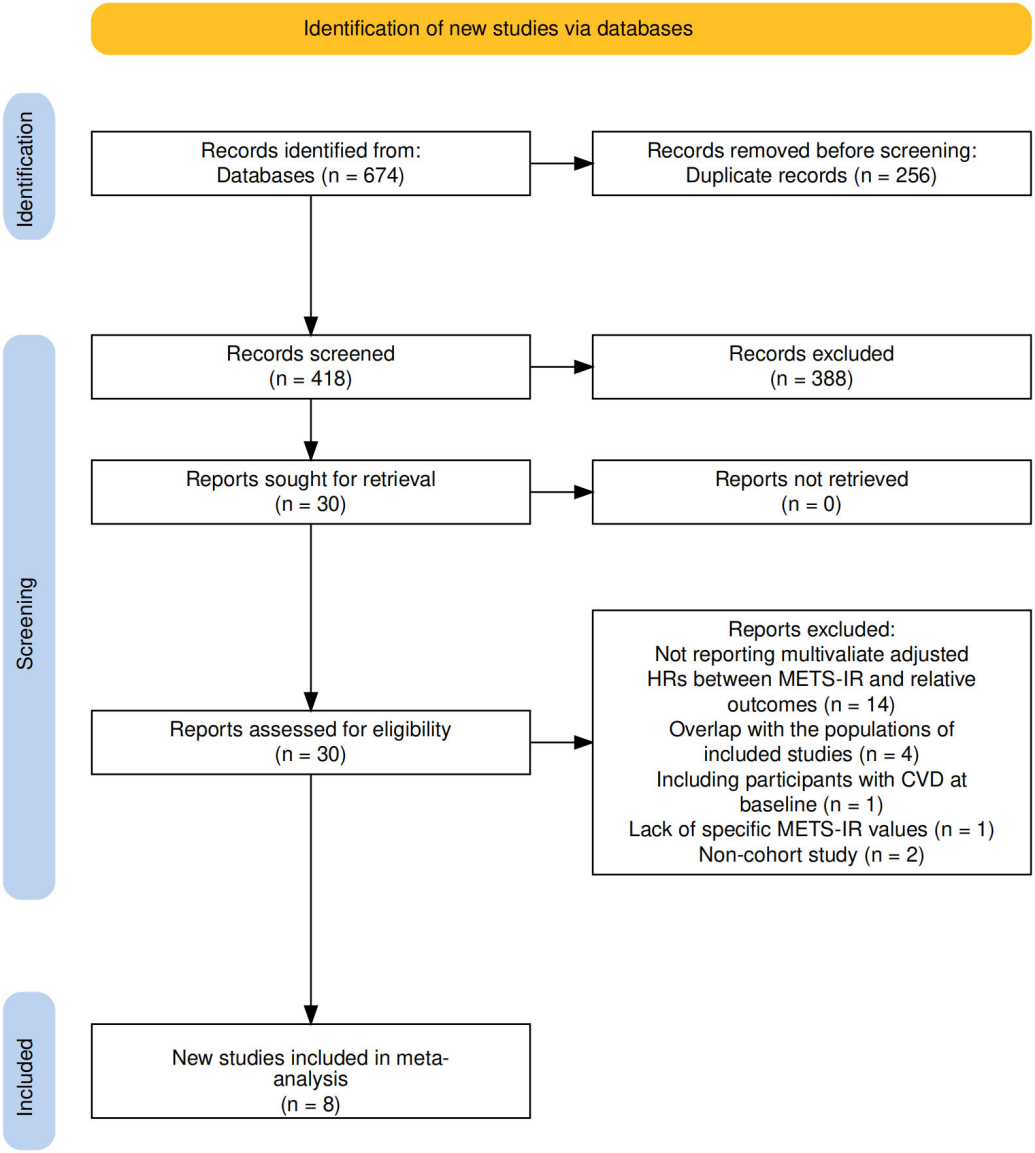
Flowchart of the database search and study identification process according to the PRISMA statement.

This study completed literature screening in accordance with the PRISMA statement (**Figure 1**). A total of 674 relevant studies were retrieved from databases (PubMed, EMBASE, The Cochrane Library, Web of Science). After removing 256 duplicate records, the remaining 418 studies underwent title and abstract screening, and 388 studies that did not meet the criteria were excluded. Through full-text assessment, additional studies were excluded for the following reasons: failure to report multivariable-adjusted HR (n=14), population overlap (n=4), inclusion of participants with baseline cardiovascular disease (CVD) (n=1), lack of specific METS-IR values (n=1), and being non-cohort studies (n=2). Finally, 8 cohort studies were included in the meta-analysis (27–34).

### Baseline characteristics of included studies

The 8 included studies (27–34) were published between 2021 and 2025 (**Table 1**), consisting of 7 prospective cohort studies (PC) (27–31, 33, 34) and 1 retrospective cohort study (RC) (32), originating from China (5 studies) (29–32, 34), South Korea (2 studies) (27, 33), and Iran (1 study) (28). The total sample size was 437,283 participants, with individual study sizes ranging from 2,031 to 306,680. All participants had no CVD at baseline (among them, Lv,2025 (34) included hypertensive participants without baseline CVD; Yang,2023 (32) included hypertensive participants with obstructive sleep apnea but without CVD; Yoon,2021 (33) included participants without diabetes or CVD). The mean age ranged from 39.4 to 59.57 years, and the male proportion was 42.9% to 71.7% (highest in Wu,2025 (31); lowest in Wang,2023 (29). Follow-up durations ranged from 1.98 to 17.9 years (shortest in Lv,2025 (34); longest in Tamehri,2024 (28). Primary outcomes included composite CVD, CAD, and stroke. Specifically, there were 317 to 5,820 composite CVD events (5 studies (29–32, 34); total 9,178 events), 198 to 1,216 CAD events (7 studies (27, 28, 30–34); total 4,579 events), and 119 to 4,659 stroke events (5 studies (28, 30–32, 34); total 6,891 events). All studies adjusted for confounders such as age, sex, smoking, alcohol consumption, hypertension, diabetes, and lipid profiles; some also adjusted for medication use (e.g., antihypertensives, hypoglycemics), physical indicators (e.g., BMI, waist circumference), and sociodemographic factors (e.g., education, marital status).

**Table 1:** Characteristics of the included cohort studies.

Study, year	Country	Design	Study population	Number of participants	Mean ages (years)	Male (%)	Mets-IR analysis	Follow-up duration (years)	Outcome validation	Outcomes reported	Variables adjusted
Ryu,2025 (27)	Korea	PC	Participants from the KoGES cohort without CVD, Participants from the HERAS–HIRA cohort without CVD	28,437	47.6	51.1	categorical; continuous	12, 4.2	ICD-10	CAD (987)	age, sex, BMI, smoking, alcohol intake, TC, eGFR, CRP, diabetes medication, hypertension medication, dyslipidemia medication, DM and mean arterial blood pressure
Tamehri,2024 (28)	Iran	PC	Community population without CVD	10,214	42.2	56.0	categorical; continuous	17.9	ICD-10	CAD(1080), stroke(267)	age, sex, smoking, diabetes, hypertension, non-HDL-C, pulse rate, serum creatinine, metabolic syndrome, lipidlowering drug use, and family history of premature CVD
Wang,2023 (29)	China	PC	Community population without CVD	4,712	39.4	42.9	categorical; continuous	5.7	ICD-10	Composite CVD(572)	age, sex, education level, exercise frequency, HDL-C, LDL-C, hypertension, family history of CVD, waist circumference, smoking and drinking
Wu,2023 (30)	China	PC	Community population without CVD	6,489	49.03	53.4	categorical; continuous	10.6	Common definitions	Composite CVD(396), CAD(247), stroke(169)	age, sex, high WHR, energy intake from fat, energy intake from carbohydrate, education, tobacco use, alcohol use, physical activity, DM, TC, hypertension, LDL-C, family history of CVD, antihypertensive drugs, and antidiabetic drugs or insulin
Wu,2025 (31)	China	PC	participants from the Kailuan Study Arterial Stiffness Subcohort without CVD	59,777	49.5	71.7	categorical; continuous	5.97	ICD-10	Composite CVD(2073), CAD(519), stroke(1677)	age, physical activity, education level, diabetes, eGFR, hypertension, sex, current smoking, BMI, current drinking and dyslipidaemia
Yang,2023 (32)	China	RC	Adults with hypertension and OSA without CVD	2,031	49.58	68.76	categorical; continuous	6.8	Medical record review	Composite CVD(317), CAD(198), stroke(119)	age, sex, drinking status, history of diabetes, DBP, SBP, eGFR, TC, LDL-C, FBG, AHI, ACEIs/ARBs, CCBs, smoking status, diuretics, OSA therapy and β-Blockers
Yoon,2021 (33)	Korea	PC	Adults without diabetes or CVD	17,943	44.7	51	categorical	2.4	ICD-10	CAD(332)	age, sex, smoking status, alcohol intake, eGFR, mean arterial blood pressure, hypertension medication, physical activity, total cholesterol and high-sensitivity C-reactive protein
Lv,2025 (34)	China	PC	Community population with hypertension and without CVD	306,680	59.57	53.48	categorical; continuous	1.98	ICD-10	Composite CVD(5820), CAD(1216), stroke(4659)	age, sex, marriage, smoke, antihypertensive drugs, antidiabetic drugs, SBP, DBP, LDL-C, education, lipid lowering drugs, exercise and drink

RC, retrospective cohort; PC, prospective cohort; BMI, body mass index; WHR, waist-to-hip ratio; eGFR, estimated glomerular filtration rate; TC, total cholesterol; HDL-C, high-density lipoprotein cholesterol; LDL-C, low-density lipoprotein cholesterol; CAD, coronary artery disease; CVD, cardiovascular disease; SBP, systolic blood pressure; DBP, diastolic blood pressure; FBG, fasting blood glucose; CRP, C-reactive protein; AHI, apnea hypopnea index; OSA, obstructive sleep apnea; DM, diabetes mellitus; ACEIs, angiotensin-converting enzyme inhibitors; ARBs, angiotensin receptor blockers; CCBs, calcium channel blockers.

### Quality assessment of included studies

Study quality was assessed using the Newcastle-Ottawa Scale (NOS) (**Table 2**). The 8 studies (27–34) scored 6 to 9 points, indicating overall high quality. Evaluations focused on population selection (representativeness, exposed/unexposed groups), group comparability (confounder adjustment), and outcome ascertainment (follow-up duration, assessment methods). Tamehri,2024 (28), Wang,2023 (29), and Wu,2023 (30) scored highest (9 points); Yoon,2021 (33) and Lv,2025 (34) scored lowest (6 points), mainly due to lower representativeness of exposed/unexposed cohorts. All studies excluded baseline outcome events and adjusted for key confounders, indicating low bias risk.

**Table 2:** Details of quality evaluation via the Newcastle–Ottawa Scale.

Study, year	Representativeness of the exposed cohort	Selection of the eon-exposed cohort	Ascertainment of exposure	Outcome not present at baseline	Comparability of cohorts on the basis of the design or analysis	Assessment of outcome	Sufficient follow-up duration	Adequacy of follow up of eohorts	Overall
Ryu,2025 (27)	1	1	1	1	2	1	1	0	8
Tamehri,2024 (28)	1	1	1	1	2	1	1	1	9
Wang,2023 (29)	1	1	1	1	2	1	1	1	9
Wu,2023 (30)	1	1	1	1	2	1	1	1	9
Wu,2025 (31)	1	1	1	1	2	1	1	0	8
Yang,2023 (32)	0	0	1	1	2	1	1	1	7
Yoon,2021 (33)	0	0	1	1	2	1	1	0	6
Lv,2025	0	0	1	1	2	1	1	0	6

### Association between METS-IR and the risk of composite CVD incidence

**Figure 2:**
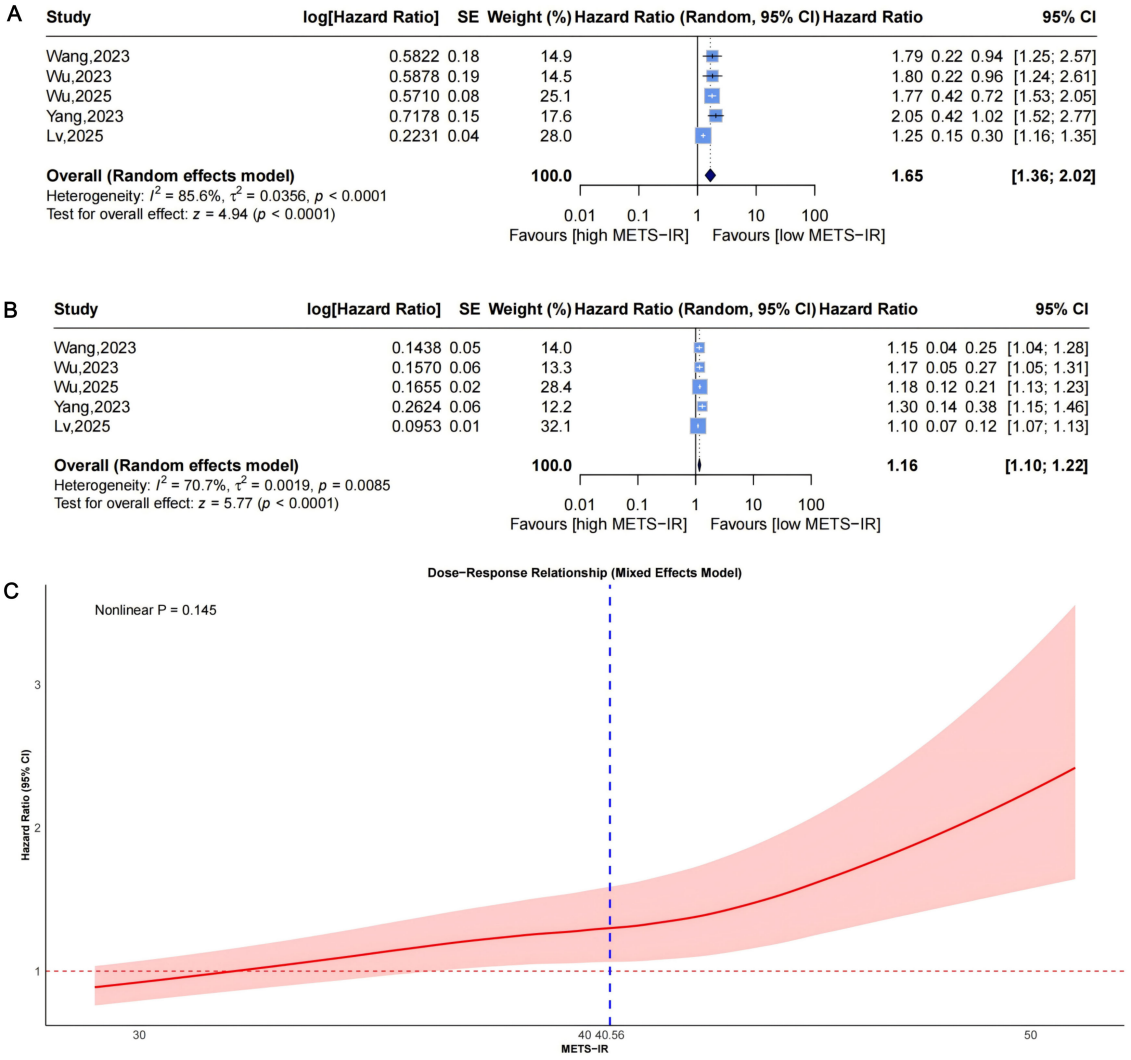
Forest plots **(A, B)** and nonlinear dose-response curve **(C)** for the association between METS-IR and CVD risk, analyzed as a categorical variable (highest vs. lowest; A) and continuous variable (per 1-SD increment; **(B)**. In forest plots, the diamond represents the pooled effect estimate; colored squares indicate study weights, and black horizontal lines denote 95% CIs of individual study effect sizes. The dose-response curve was fitted using restricted cubic spline regression; the red line shows the pooled association, with the red shaded area representing 95% CIs. The blue dashed line marks the key inflection point (METS-IR = 40.56). METS-IR, metabolic score for insulin resistance; CVD, cardiovascular disease; CI, confidence interval; SD, standard deviation.

A random-effects model pooled effect sizes from 5 studies (**Figure 2A**), showing that the highest baseline METS-IR group had an increased CVD risk compared to the lowest (HR = 1.65, 95% CI: 1.36-2.02, I²=85.6%, τ²=0.0356, P<0.0001). This was aligned with the continuous analysis (5 studies; per 1-SD increase: HR = 1.16, 95% CI: 1.10-1.22, I²=70.7%, τ²=0.0019, P<0.0001, **Figure 2B**).

**Figure 3:**
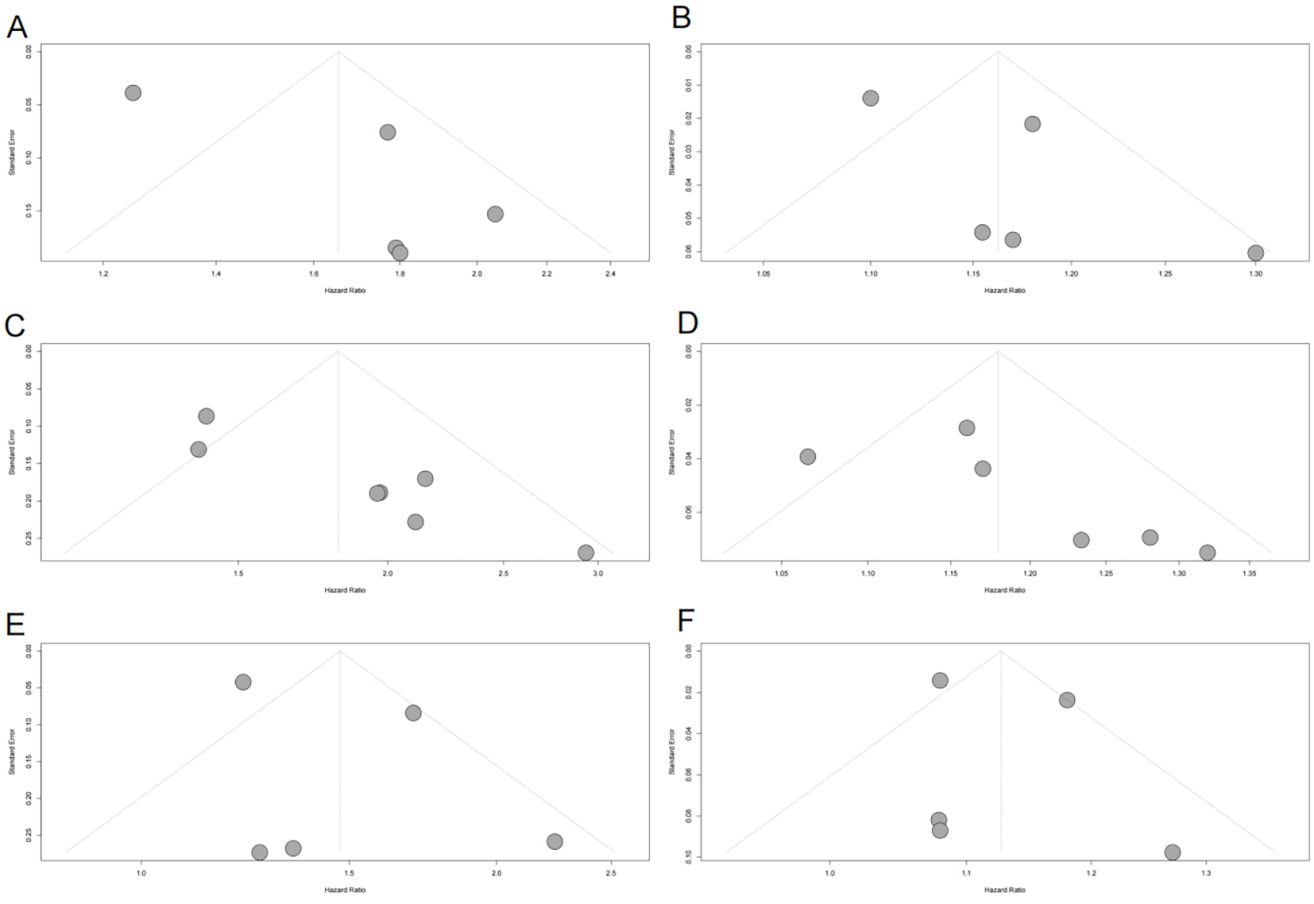
Funnel plots assessing publication bias in the meta-analysis of the association between METS-IR and CVD, CAD, and stroke risks. **(A)** METS-IR as a categorical variable and CVD risk; **(B)** METS-IR as a continuous variable and CVD risk; **(C)** METS-IR as a categorical variable and CAD risk; **(D)** METS-IR as a continuous variable and CAD risk; **(E)** METS-IR as a categorical variable and stroke risk; **(F)** METS-IR as a continuous variable and stroke risk. Gray dots represent individual included studies. METS-IR, metabolic score for insulin resistance; CVD, cardiovascular disease; CAD, coronary artery disease.

Funnel plots indicated asymmetry for both categorical (**Figure 3A**, trim-and-fill estimated 2 missing studies, SE = 1.6604; adjusted HR = 1.55, 95% CI: 1.28-1.86, **Supplementary Material: Figure S1A**, **4A**) and continuous analyses (**Figure 3B**, 3 missing studies, SE = 1.4845; adjusted HR = 1.11, 95% CI: 1.05-1.17; **Supplementary Material: Figures S1B**, **4B**), suggesting potential missing negative-result studies. Sensitivity analyses confirmed robust results: categorical HR ranged from 1.58-1.82; continuous HR ranged from 1.16-1.17 (all P<0.0001; **Figures 5A, B**). Omitting Lv,2025 (34) led to the most pronounced I² reductions (e.g., to 0% for composite CVD; **Figures 5**–**7**). Due to fewer than 10 studies, Egger’s test and subgroup analyses were not performed.

**Figure 4:**
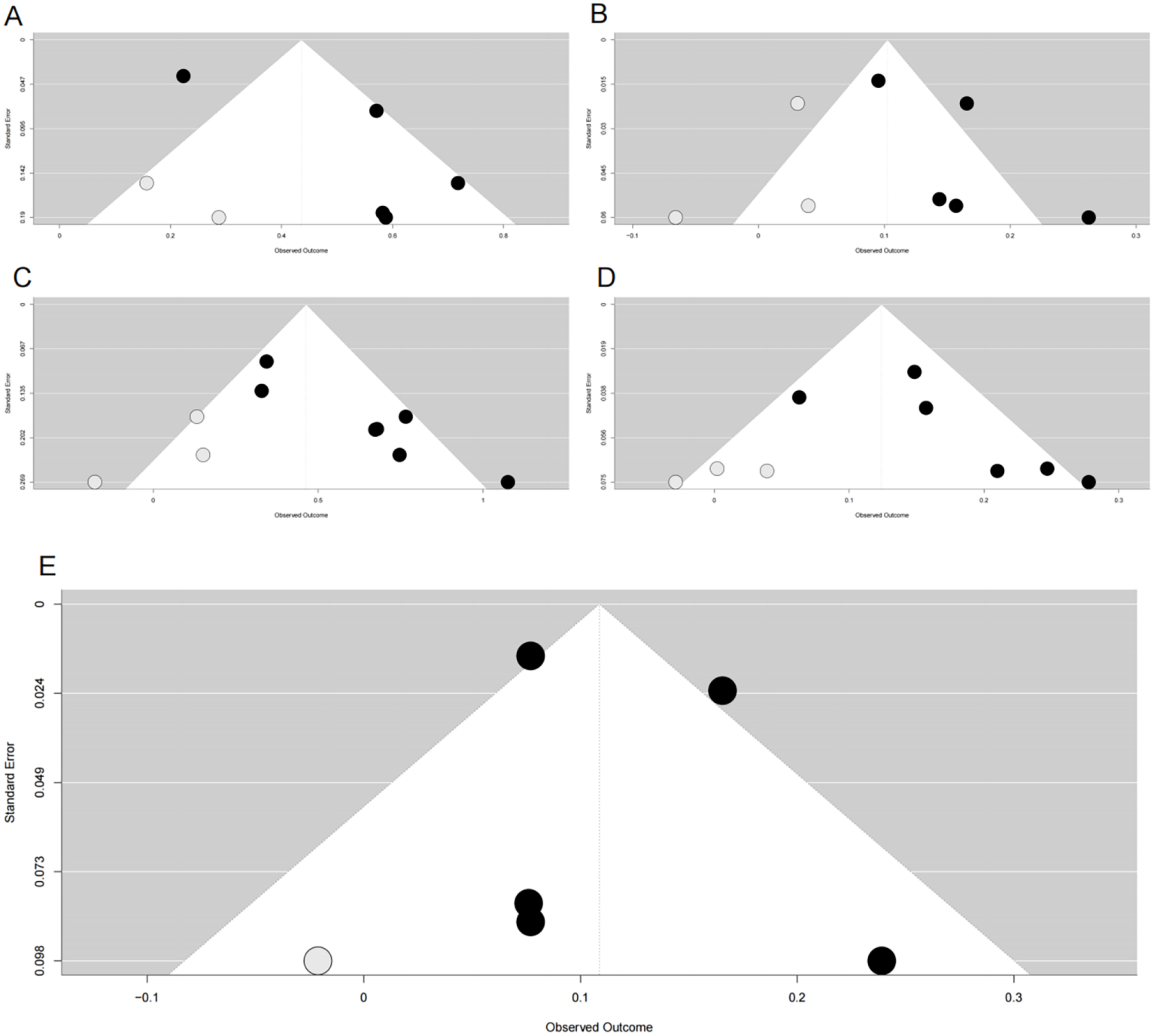
Funnel plots after trim-and-fill adjustment for publication bias in associations between METS-IR and cardiovascular risks. **(A)** METS-IR as a categorical variable vs. CVD risk; **(B)** METS-IR as a continuous variable vs. CVD risk; **(C)** METS-IR as a categorical variable vs. CAD risk; **(D)** METS-IR as a continuous variable vs. CAD risk; **(E)** METS-IR as a continuous variable vs. stroke risk. Black dots represent originally included studies; gray dots represent studies imputed by the trim-and-fill method. METS-IR, metabolic score for insulin resistance; CVD, cardiovascular disease; CAD, coronary artery disease.

**Figure 5:**
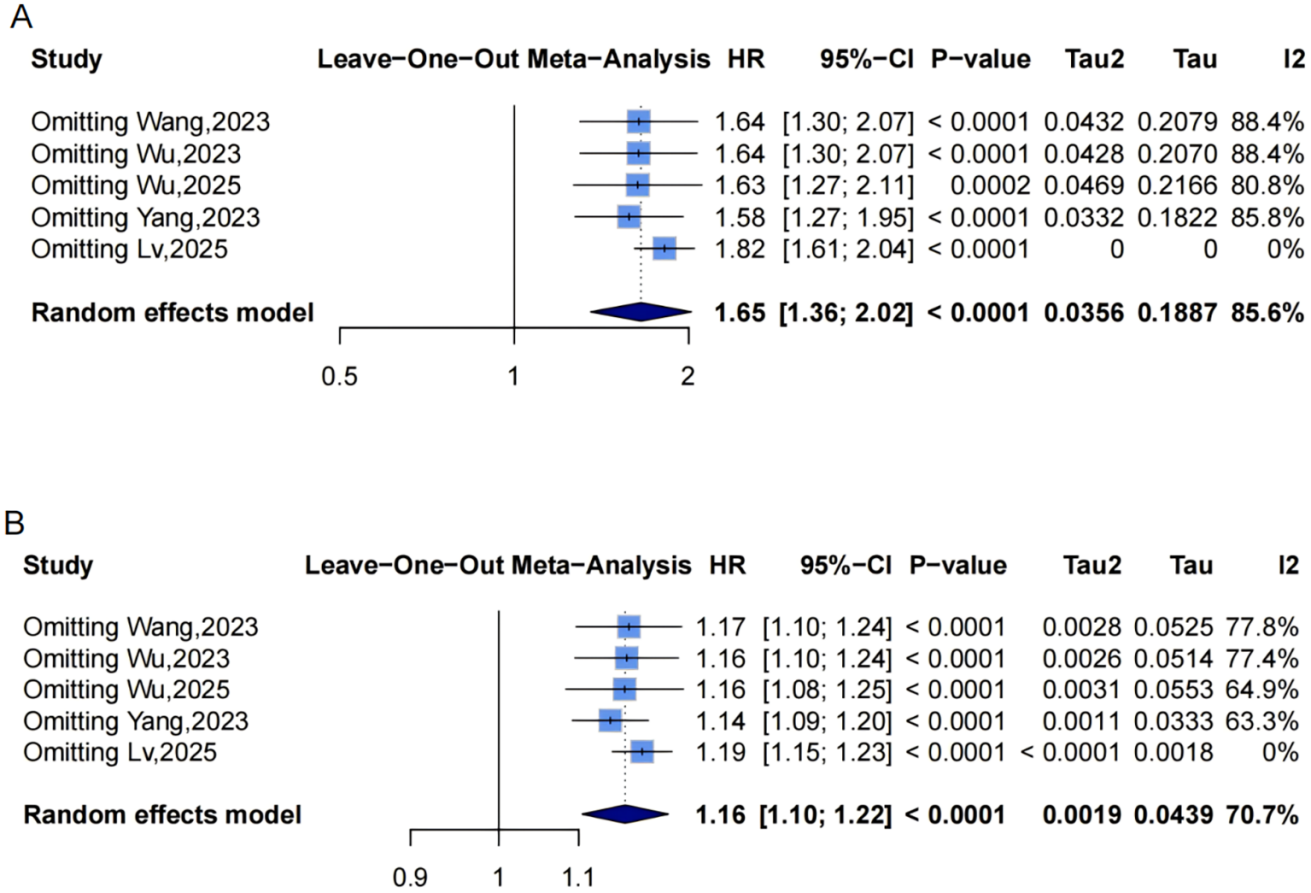
Leave-one-out sensitivity analyses of the meta-analysis for METS-IR and CVD risk. Blue squares denote the pooled HR for each leave-one-out scenario, with horizontal lines indicating 95% CIs. The diamond at the bottom represents the overall pooled HR from the random-effects model. **(A)** METS-IR as a categorical variable; **(B)** METS-IR as a continuous variable. METS-IR, metabolic score for insulin resistance; CVD, cardiovascular disease; HR, hazard ratio; CI, confidence interval.

**Figure 6:**
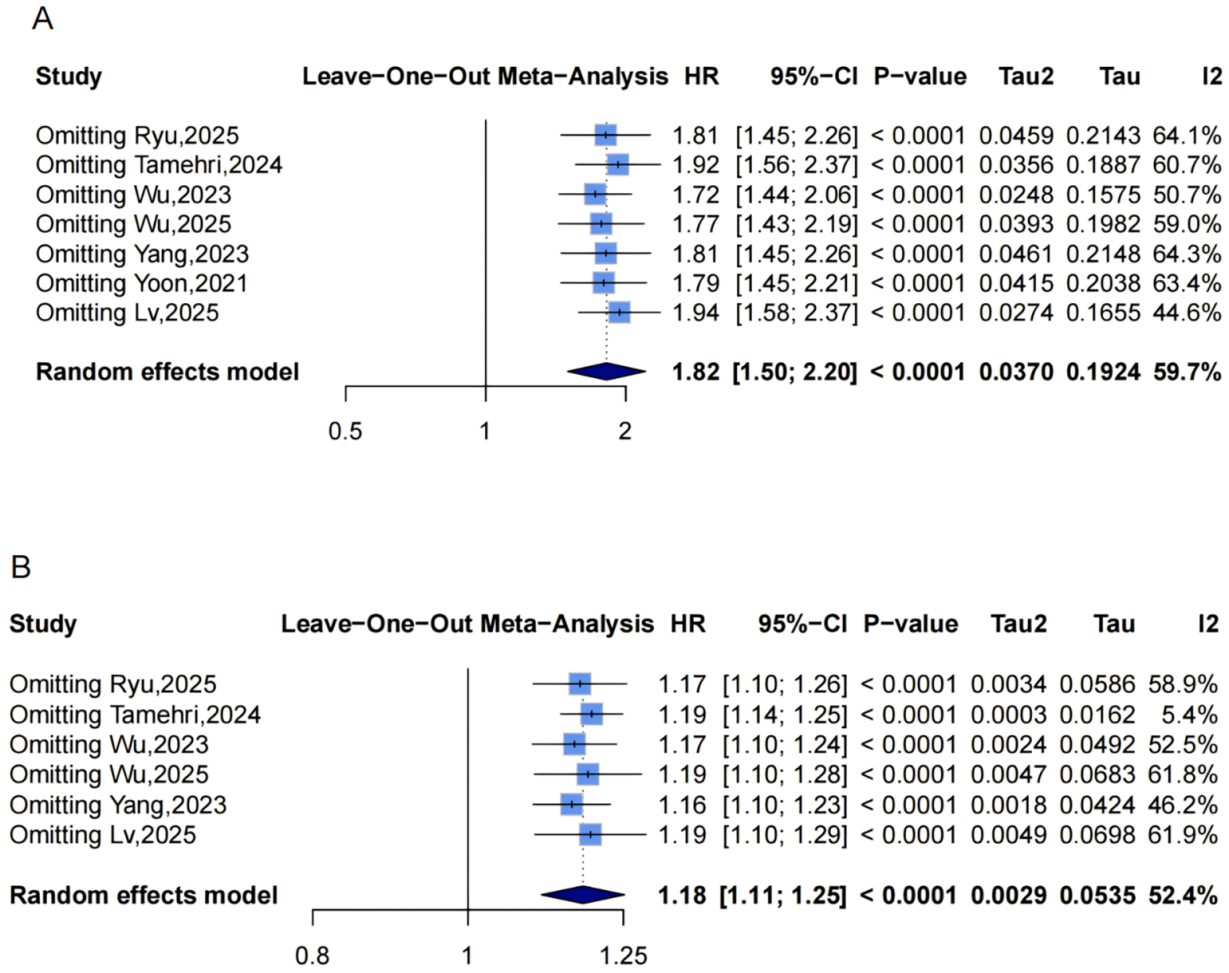
Leave-one-out sensitivity analyses of the meta-analysis for METS-IR and CAD risk. Blue squares denote the pooled HR for each leave-one-out scenario, with horizontal lines indicating 95% CIs. The diamond at the bottom represents the overall pooled HR from the random-effects model. **(A)** METS-IR as a categorical variable; **(B)** METS-IR as a continuous variable. METS-IR, metabolic score for insulin resistance; CAD, coronary artery disease; HR, hazard ratio; CI, confidence interval.

**Figure 7:**
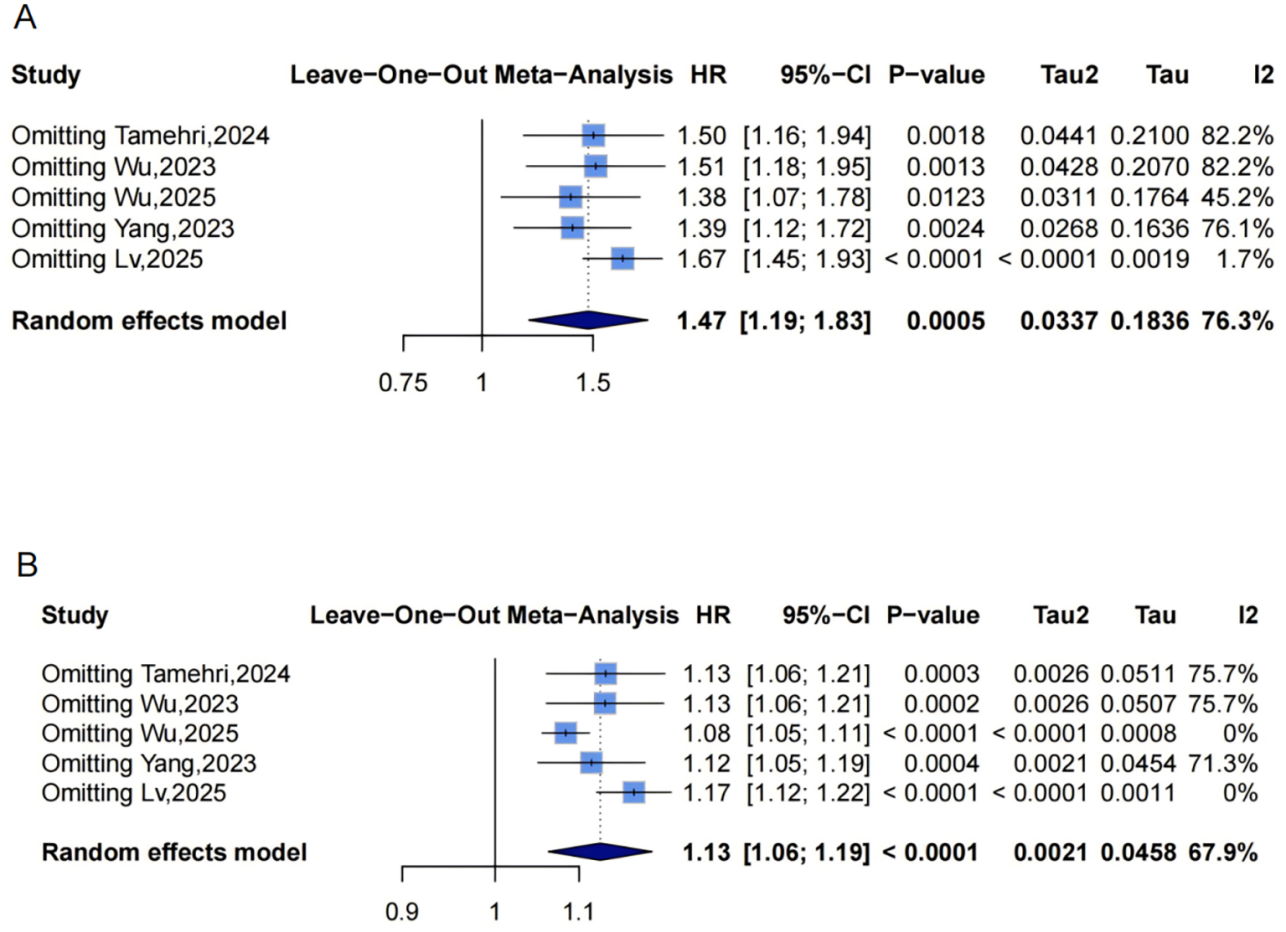
Leave-one-out sensitivity analyses of the meta-analysis for METS-IR and stroke risk. Blue squares denote the pooled HR for each leave-one-out scenario, with horizontal lines indicating 95% CIs. The diamond at the bottom represents the overall pooled HR from the random-effects model. **(A)** METS-IR as a categorical variable; **(B)** METS-IR as a continuous variable. METS-IR, metabolic score for insulin resistance; HR, hazard ratio; CI, confidence interval.

Dose-response analysis using a mixed-effects model with restricted cubic splines (P for nonlinearity =0.145, **Figure 2C**) did not show statistically significant nonlinearity but suggested a potential pattern: HR remained near 1 at low METS-IR, rising with increases and widening CIs. An exploratory inflection point was identified at approximately 40.56, beyond which risk appeared to accelerate.

### Association between METS-IR and the risk of coronary artery disease incidence

**Figure 8:**
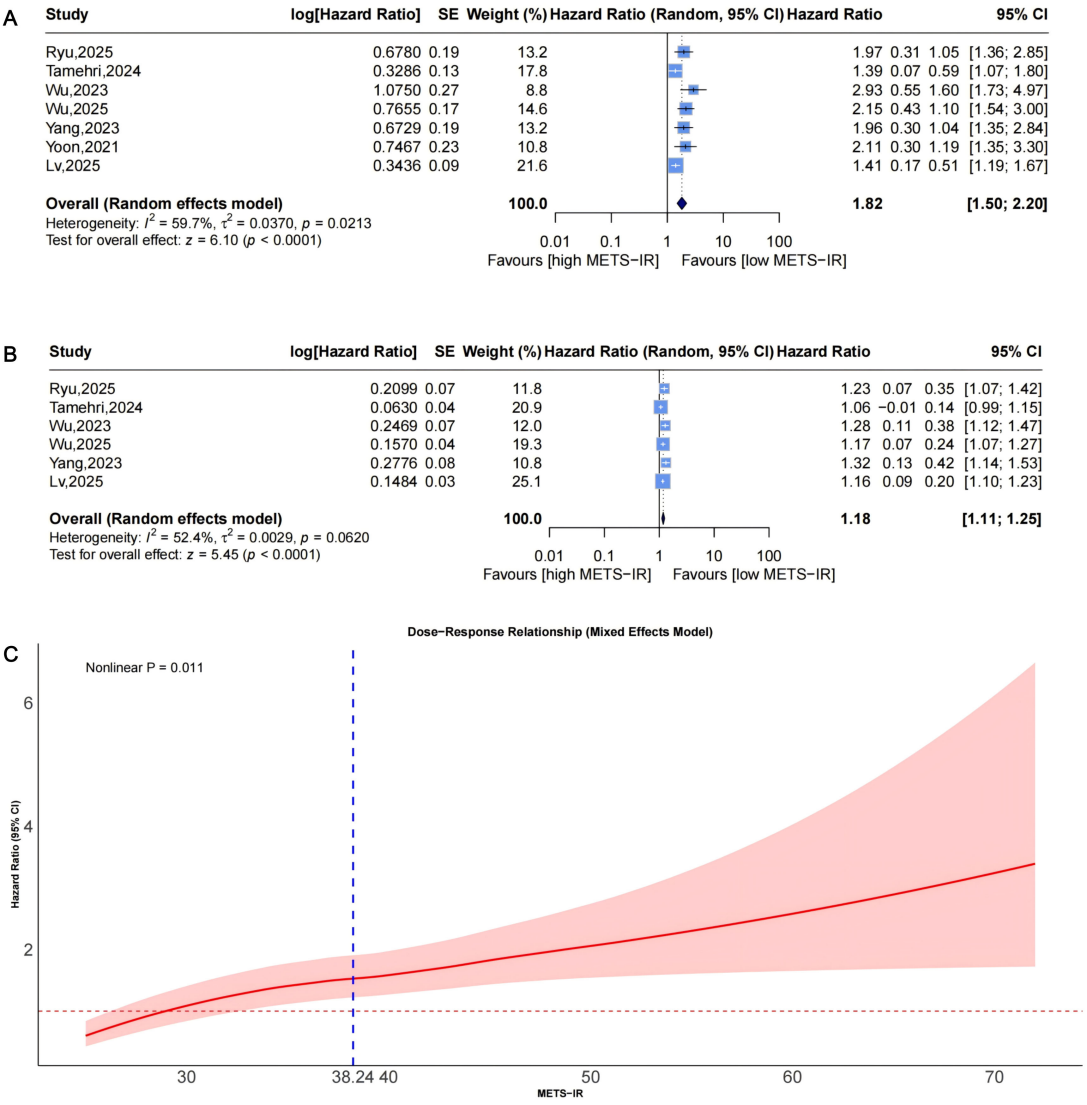
Forest plots **(A, B)** and nonlinear dose-response curve **(C)** for the association between METS-IR and CAD risk, analyzed as a categorical variable (highest vs. lowest; **(A)** and continuous variable (per 1-SD increment; **(B)**. In forest plots, the diamond represents the pooled effect estimate; colored squares indicate study weights, and black horizontal lines denote 95% CIs of individual study effect sizes. The dose-response curve was fitted using restricted cubic spline regression; the red line shows the pooled association, with the red shaded area representing 95% CIs. The blue dashed line marks the key inflection point (METS-IR = 38.24). METS-IR, metabolic score for insulin resistance; CAD, coronary artery disease; CI, confidence interval; SD, standard deviation.

Pooled results from 7 studies (**Figure 8A**) indicated a higher CAD risk in the highest METS-IR group (HR = 1.82, 95% CI: 1.50-2.20, I²=59.7%, τ²=0.0370, P<0.0001). The continuous analysis (6 studies; per 1-SD: HR = 1.18, 95% CI: 1.11-1.25, I²=52.4%, τ²=0.0029, P<0.0001, **Figure 8B**) was consistent.

Funnel plots showed asymmetry for both categorical (**Figure 3C**, 3 missing studies, SE = 1.6850, adjusted HR = 1.59, 95% CI: 1.32-1.92, **Supplementary Material: Figures S2A**, **4C**) and continuous analyses (**Figure 3D**, 3 missing studies, SE = 1.6385; adjusted HR = 1.13, 95% CI: 1.07-1.20; **Supplementary Material: Figures S2B**, **4D**). Sensitivity analyses showed stable results: categorical HR ranged from 1.72-1.94; continuous HR ranged from 1.16-1.19 (all P<0.0001, **Figures 6A, B**). Omitting Lv,2025 (34) yielded the most pronounced I² reductions (e.g., from 59.7% to 44.6% for CAD categorical analysis, **Figures 5**–**7**). Fewer than 10 studies precluded further tests.

Dose-response analysis (P for nonlinearity=0.011; **Figure 8C**) exhibited nonlinearity: HR was near 1 at low levels, with an upward trend and widening CIs. The inflection point was at approximately 38.24, indicating accelerated risk beyond this threshold.

### Association between METS-IR and the risk of stroke incidence

**Figure 9:**
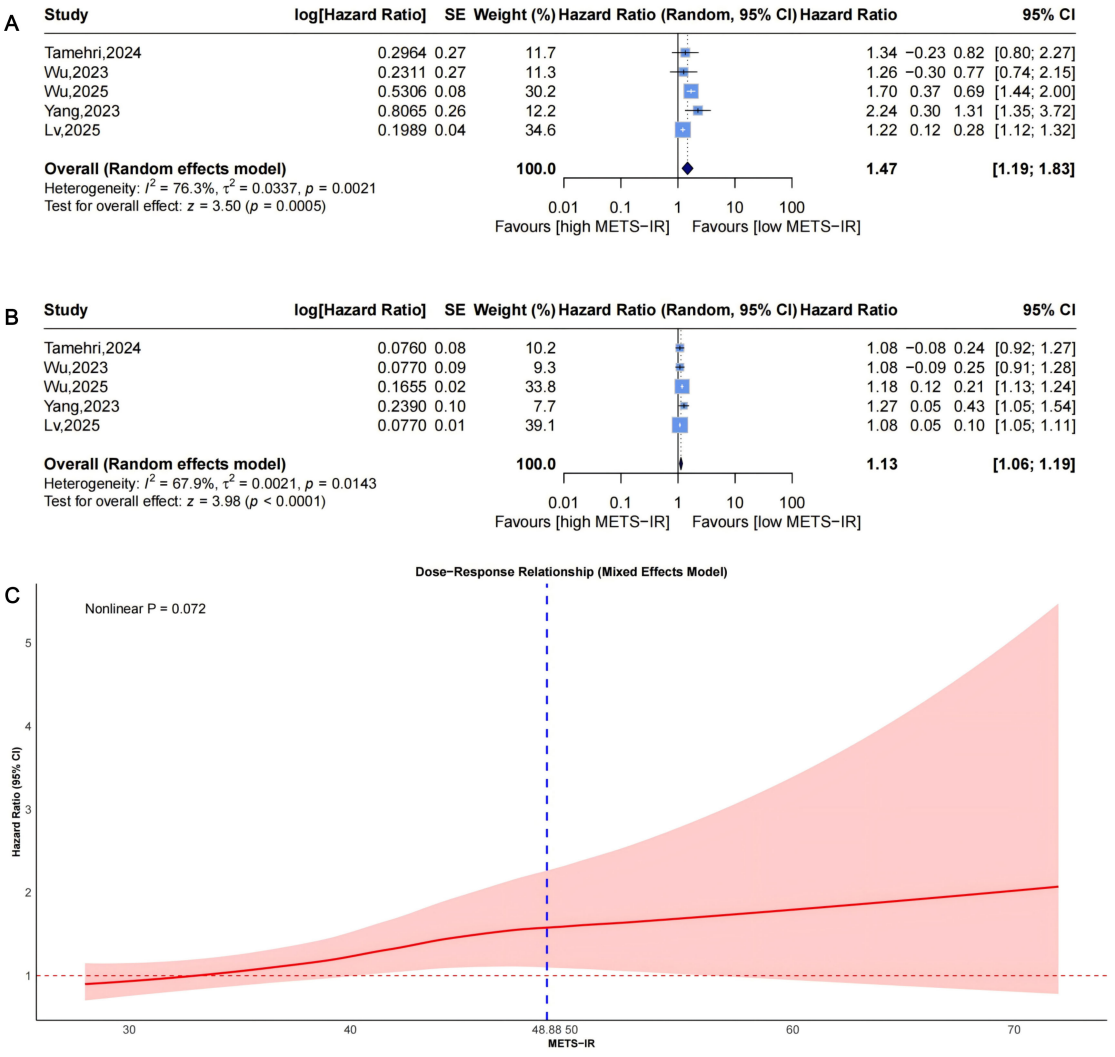
Forest plots **(A, B)** and nonlinear dose-response curve **(C)** for the association between METS-IR and stroke risk, analyzed as a categorical variable (highest vs. lowest; **(A)** and continuous variable (per 1-SD increment; **(B)**. In forest plots, the diamond represents the pooled effect estimate; colored squares indicate study weights, and black horizontal lines denote 95% CIs of individual study effect sizes. The dose-response curve was fitted using restricted cubic spline regression; the red line shows the pooled association, with the red shaded area representing 95% CIs. The blue dashed line marks the key inflection point (METS-IR = 48.88). METS-IR, metabolic score for insulin resistance; CI, confidence interval; SD, standard deviation.

Pooled from 5 studies (**Figure 9A**) indicated that the highest METS-IR increased stroke risk (HR = 1.47, 95% CI: 1.19-1.83, I²=76.3%, τ²=0.0337, P = 0.0005). The continuous analysis (5 studies; per 1-SD: HR = 1.13, 95% CI: 1.06-1.19, I²=67.9%, τ²=0.0021, P<0.0001, **Figure 9B**) aligned with this finding.

Funnel plots indicated categorical symmetry (**Figure 3E**, 0 missing studies; **Supplementary Material: Figure S3A**) but continuous asymmetry (**Figure 3F**, 1 missing study, SE = 1.7124; adjusted HR = 1.11, 95% CI: 1.05-1.19; **Supplementary Material: Figures S3B**, **4E**). Sensitivity analyses confirmed robustness: categorical HR ranged from 1.38-1.67; continuous HR ranged from 1.08-1.17 (all P<0.05, **Figures 7A, B**). Omittig Lv,2025 (34) led to the most pronounced I² reductions (e.g., to 0% for stroke continuous analysis, **Figures 5**–**7**). Due to fewer than 10 studies, Egger’s test and subgroup analyses were omitted.

Dose-response analysis (P for nonlinearity=0.072, **Figure 9C**) showed marginal nonlinearity: HR was approximately 1 at low METS-IR levels, with an upward trend and widening CIs. The inflection point was at approximately 48.88, beyond which risk accelerated.

## Discussion

### Main findings

The main findings of this meta-analysis are that higher METS-IR levels are significantly associated with increased risks of composite CVD, CAD, and stroke in adults without baseline CVD. Specifically, the pooled hazard ratios (HRs) for the highest versus lowest METS-IR categories were 1.65 (95% CI: 1.36-2.02) for composite CVD, 1.82 (1.50-2.20) for CAD, and 1.47 (1.19-1.83) for stroke. Dose-response analyses further revealed nonlinear relationships for CAD (P = 0.011; inflection at approximately 38.24), marginal nonlinearity for stroke (P = 0.072; inflection at approximately 48.88), and a potential nonlinear pattern for composite CVD (P = 0.145; exploratory inflection at approximately 40.56), beyond which risks accelerate. These results were consistent across categorical and continuous analyses, with moderate-to-high heterogeneity (I²=52.4%-85.6%).

### Comparison with other studies

These findings align with prior meta-analyses on other insulin resistance surrogates, such as HOMA-IR and TyG, which also demonstrate independent predictive value for CVD. For instance, a meta-analysis of 65 studies involving over 500,000 participants without diabetes reported that HOMA-IR was associated with a higher risk of coronary heart disease (HR = 1.46 per SD) compared to glucose (HR = 1.21) or insulin (HR = 1.04) alone (35). This is similar to our observed HR of 1.18 per SD for METS-IR and CAD. Additionally, a study of HOMA-IR trajectories in 6,755 Koreans showed that increasing patterns over approximately 5 years elevated CVD incidence (HR = 1.59) and mortality (HR = 2.33) (36), complementing our nonlinear dose-response curves. Compared to TyG, METS-IR’s HR for CAD (1.82) is comparable to reported values [2.01 (37); 1.94 (8)], though slightly lower, possibly due to our focus on Asian cohorts or differences in adjustments. For composite CVD, METS-IR’s HR (1.65) matches that of TyG-BMI’s [1.62 (9)], indicating equivalent predictive utility despite METS-IR’s simpler components.

### Biological mechanisms

IR, often accompanied by compensatory hyperinsulinemia, serves as an independent risk factor for numerous diseases, including type 2 diabetes, CVD, cellular senescence, tumors, and neurodegenerative disorders (2–4, 38, 39). Specifically, in the context of CVD, IR and hyperinsulinemia contribute to vascular and myocardial damage through several interconnected mechanisms. IR impairs endothelial function by reducing nitric oxide bioavailability, promoting oxidative stress, and activating pro-inflammatory pathways such as NF-κB, leading to endothelial dysfunction, inflammation, and accelerated atherosclerosis (3, 4, 40). Hyperinsulinemia exacerbates these effects by stimulating vascular smooth muscle cell proliferation, migration, and extracellular matrix deposition, which fosters plaque formation and vascular stiffness (40). Additionally, in the myocardium, IR disrupts fatty acid metabolism, inducing lipotoxicity, mitochondrial dysfunction, and increased susceptibility to ischemia, contributing to diabetic cardiomyopathy and heart failure (41–43). These pathophysiological processes form the basis of the association between elevated METS-IR, as a surrogate marker of IR, and the increased incidence of composite CVD, CAD, and stroke observed in our meta-analysis. Observed heterogeneity (I²=52.4%-85.6%) likely stems from study diversity, including baseline characteristics (e.g., hypertension, non-diabetes), follow-up durations (1.98-17.9 years), and geography (e.g., longer follow-up in Tamehri et al. (28)amplifying cumulative effects). The nonlinear patterns suggest threshold effects, where low METS-IR yields gradual risk increases, but exceeding inflections amplifies oxidative stress and lipotoxicity, driving sharper CVD escalation (41–43).

### Strengths

One key strength of this meta-analysis is that it represents the first comprehensive summary of the link between METS-IR and incident CVD. We pooled data from eight high-quality cohort studies (NOS scores: 6-9; total N = 437,283) from China, Korea, and Iran, all of which adjusted for major confounders like age, sex, smoking, hypertension, diabetes, and lipids profiles. Our use of advanced techniques, like restricted cubic splines for dose-response modeling, provides detailed thresholds that could prove useful in clinical settings. The results held up well in sensitivity analyses (with stable HRs) and trim-and-fill adjustments (maintaining significance even after accounting for potential missing studies), despite some heterogeneity.

### Limitations

This meta-analysis, while robust, has several limitations that warrant consideration. First, with only eight studies included, despite the large overall sample size—we could not perform subgroup analyses, as our predefined criteria required at least 10 studies. Second, we detected possible publication bias in the analyses for composite CVD and CAD using trim-and-fill methods, which suggested 2–3 missing studies with null results; while sensitivity tests showed the findings were robust, the actual effect sizes might be slightly smaller (e.g., adjusted HRs: 1.55 [95% CI: 1.28-1.86] for composite CVD; 1.59 [1.32-1.92] for CAD). Third, differences in how METS-IR was categorized, and endpoints were defined (e.g., ICD-10 codes vs. medical records) may have affected the pooled estimates, though we addressed this with random-effects models and sensitivity checks. The moderate-to-high heterogeneity (I²=52.4%-85.6%) across outcomes probably arises from variations in follow-up length, geographic settings, and baseline participant risks; for example, excluding Lv et al. (2025) (34)—which had the shortest follow-up at 1.98 years—sharply lowered I² (e.g., from 85.6% to 0% for composite CVD categorical analysis and 67.9% to 0% for stroke continuous analysis), since shorter studies might miss longer-term effects and increase type II error risk (44). Fourth, the observational nature of the cohorts limits our ability to infer causality, as unmeasured confounders like genetics could play a role. Finally, since most cohorts were from Asia, the results may not generalize well, calling for replication in African, European, and Latino populations.

### Implications and future directions

Despite these limitations, our findings carry important implications for clinical practice and public health. METS-IR stands out as an easy-to-use biomarker for assessing CVD risk in adults without symptoms, outperforming some traditional IR measures because it draws on standard lab values (7). With HRs like 1.65 for composite CVD, 1.82 for CAD, and 1.47 for stroke (comparing highest to lowest categories), it could help spot high-risk individuals early, especially those with hypertension or other metabolic issues—and steer them toward lifestyle tweaks or medications to stay below key inflection points (11, 32, 45). On a broader scale, its simple, low-cost nature makes it ideal for widespread screening in areas where advanced tests aren’t feasible (7, 12, 46, 47), fitting well with worldwide efforts to prevent CVD through better metabolic control (10, 48, 49). To build on this, future studies should test these associations in more diverse groups, such as non-Asian ethnicities or younger adults, to improve generalizability and refine the inflection points. We also need mechanistic research to unpack the nonlinear patterns—for CAD, stroke, and composite CVD—by examining how METS-IR’s elements (like BMI, fasting blood glucose, and TG/HDL-C) interact with factors such as endothelial dysfunction or inflammation. Head-to-head comparisons with other surrogates, like the TyG index, could reveal if METS-IR adds unique value in combined models. Finally, long-term trials that lower METS-IR through diet or exercise would help establish causality and quantify how much risk can be reduced.

## Conclusion

In summary, this meta-analysis demonstrates that higher METS-IR is significantly associated with increased risks of composite CVD, CAD, and stroke, with nonlinear dose-response relationships for CAD, marginal nonlinear dose-response relationships for stroke, and a potential nonlinear dose-response relationship for composite CVD, including critical inflection points beyond which risk accelerates. These findings validate METS-IR as a valuable tool for cardiovascular risk assessment and provide practical thresholds for clinical practice. Despite limitations, the consistency of results across large, well-designed cohort studies supports the utility of METS-IR in guiding preventive strategies for cardiovascular disease.

## Data Availability

Publicly available datasets were analyzed in this study. This data can be found here: The datasets analyzed in this meta-analysis are derived from the following publicly available studies cited in the article: Qian T, et al. (2023) doi: 10.3389/fendo.2023.1224967;Tazeem MS, et al. (2024) doi: 10.7759/cureus.70289; and others listed in References 11-15, 27-34. No centralized repository or accession numbers are applicable; data are accessible via the respective journal articles.
