# Supplementary material for: Metabolic score for insulin resistance and the incidence of cardiovascular disease: a meta-analysis of cohort studies
Source: Front Endocrinol (Lausanne). 2025 Oct 17;16:1699985. doi: 10.3389/fendo.2025.1699985 (PMC12575141; doi:10.3389/fendo.2025.1699985)
Supplement: Supplementary file 1 [file DataSheet1.docx]

**Table S1. PRISMA 2020 Checklist**

| **Section and Topic** | **Item #** | **Checklist item** | **Location where item is reported** |
| --- | --- | --- | --- |
| **TITLE** | | |  |
| Title | 1 | Identify the report as a systematic review. | 1 |
| **ABSTRACT** | | |  |
| Abstract | 2 | See the PRISMA 2020 for Abstracts checklist. | 2 |
| **INTRODUCTION** | | |  |
| Rationale | 3 | Describe the rationale for the review in the context of existing knowledge. | 3-4 |
| Objectives | 4 | Provide an explicit statement of the objective(s) or question(s) the review addresses. | 4 |
| **METHODS** | | |  |
| Eligibility criteria | 5 | Specify the inclusion and exclusion criteria for the review and how studies were grouped for the syntheses. | 6 |
| Information sources | 6 | Specify all databases, registers, websites, organisations, reference lists and other sources searched or consulted to identify studies. Specify the date when each source was last searched or consulted. | 5 |
| Search strategy | 7 | Present the full search strategies for all databases, registers and websites, including any filters and limits used. | Table S2 |
| Selection process | 8 | Specify the methods used to decide whether a study met the inclusion criteria of the review, including how many reviewers screened each record and each report retrieved, whether they worked independently, and if applicable, details of automation tools used in the process. | 6 |
| Data collection process | 9 | Specify the methods used to collect data from reports, including how many reviewers collected data from each report, whether they worked independently, any processes for obtaining or confirming data from study investigators, and if applicable, details of automation tools used in the process. | 7 |
| Data items | 10a | List and define all outcomes for which data were sought. Specify whether all results that were compatible with each outcome domain in each study were sought (e.g. for all measures, time points, analyses), and if not, the methods used to decide which results to collect. | 6-7 |
|  | 10b | List and define all other variables for which data were sought (e.g. participant and intervention characteristics, funding sources). Describe any assumptions made about any missing or unclear information. | 7 |
| Study risk of bias assessment | 11 | Specify the methods used to assess risk of bias in the included studies, including details of the tool(s) used, how many reviewers assessed each study and whether they worked independently, and if applicable, details of automation tools used in the process. | 7, Table 2 |
| Effect measures | 12 | Specify for each outcome the effect measure(s) (e.g. risk ratio, mean difference) used in the synthesis or presentation of results. | 7-8 |
| Synthesis methods | 13a | Describe the processes used to decide which studies were eligible for each synthesis (e.g. tabulating the study intervention characteristics and comparing against the planned groups for each synthesis (item #5)). | 6 |
|  | 13b | Describe any methods required to prepare the data for presentation or synthesis, such as handling of missing summary statistics, or data conversions. | 7-8 |
|  | 13c | Describe any methods used to tabulate or visually display results of individual studies and syntheses. | 7-8 |
|  | 13d | Describe any methods used to synthesize results and provide a rationale for the choice(s). If meta-analysis was performed, describe the model(s), method(s) to identify the presence and extent of statistical heterogeneity, and software package(s) used. | 8 |
|  | 13e | Describe any methods used to explore possible causes of heterogeneity among study results (e.g. subgroup analysis, meta-regression). | 8 |
|  | 13f | Describe any sensitivity analyses conducted to assess robustness of the synthesized results. | 8 |
| Reporting bias assessment | 14 | Describe any methods used to assess risk of bias due to missing results in a synthesis (arising from reporting biases). | 8 |
| Certainty assessment | 15 | Describe any methods used to assess certainty (or confidence) in the body of evidence for an outcome. | 7 |
| **RESULTS** | | |  |
| Study selection | 16a | Describe the results of the search and selection process, from the number of records identified in the search to the number of studies included in the review, ideally using a flow diagram. | 9, Fig. 1 |
|  | 16b | Cite studies that might appear to meet the inclusion criteria, but which were excluded, and explain why they were excluded. | 9 |
| Study characteristics | 17 | Cite each included study and present its characteristics. | 9-10, Table 1 |
| Risk of bias in studies | 18 | Present assessments of risk of bias for each included study. | 10, Table 2 |
| Results of individual studies | 19 | For all outcomes, present, for each study: (a) summary statistics for each group (where appropriate) and (b) an effect estimate and its precision (e.g. confidence/credible interval), ideally using structured tables or plots. | 10-11, Figs. 2-4 |
| Results of syntheses | 20a | For each synthesis, briefly summarise the characteristics and risk of bias among contributing studies. | 9-10 |
|  | 20b | Present results of all statistical syntheses conducted. If meta-analysis was done, present for each the summary estimate and its precision (e.g. confidence/credible interval) and measures of statistical heterogeneity. If comparing groups, describe the direction of the effect. | 10-11, Figs. 2-4 |
|  | 20c | Present results of all investigations of possible causes of heterogeneity among study results. | 6, 11 |
|  | 20d | Present results of all sensitivity analyses conducted to assess the robustness of the synthesized results. | 6, 11, Figs. 7-9 |
| Reporting biases | 21 | Present assessments of risk of bias due to missing results (arising from reporting biases) for each synthesis assessed. | 6, 11, Figs. 5-6, S1-S3 |
| Certainty of evidence | 22 | Present assessments of certainty (or confidence) in the body of evidence for each outcome assessed. | 10–11 |
| **DISCUSSION** | | |  |
| Discussion | 23a | Provide a general interpretation of the results in the context of other evidence. | 11-13 |
|  | 23b | Discuss any limitations of the evidence included in the review. | 13-14 |
|  | 23c | Discuss any limitations of the review processes used. | 13-14 |
|  | 23d | Discuss implications of the results for practice, policy, and future research. | 14 |
| **OTHER INFORMATION** | | |  |
| Registration and protocol | 24a | Provide registration information for the review, including register name and registration number, or state that the review was not registered. | 5 |
|  | 24b | Indicate where the review protocol can be accessed, or state that a protocol was not prepared. | 5 |
|  | 24c | Describe and explain any amendments to information provided at registration or in the protocol. | Not reported |
| Support | 25 | Describe sources of financial or non-financial support for the review, and the role of the funders or sponsors in the review. | 15 |
| Competing interests | 26 | Declare any competing interests of review authors. | 15 |
| Availability of data, code and other materials | 27 | Report which of the following are publicly available and where they can be found: template data collection forms; data extracted from included studies; data used for all analyses; analytic code; any other materials used in the review. | 15 |

*From:*  Page MJ, McKenzie JE, Bossuyt PM, Boutron I, Hoffmann TC, Mulrow CD, et al. The PRISMA 2020 statement: an updated guideline for reporting systematic reviews. BMJ 2021;372:n71. doi: 10.1136/bmj.n71. This work is licensed under CC BY 4.0. To view a copy of this license, visit <https://creativecommons.org/licenses/by/4.0/>

**Table S2:** Detailed description of the search strategy

| **PubMed** | |
| --- | --- |
| #1 | Cardiovascular Diseases[Mesh] |
| #2 | (((((((((Cardiovascular Disease[Title/Abstract]) OR (Disease, Cardiovascular[Title/Abstract])) OR (Cardiac Events[Title/Abstract])) OR (Cardiac Event[Title/Abstract])) OR (Event, Cardiac[Title/Abstract])) OR (Adverse Cardiac Event[Title/Abstract])) OR (Adverse Cardiac Events[Title/Abstract])) OR (Cardiac Event, Adverse[Title/Abstract])) OR (Cardiac Events, Adverse[Title/Abstract])) OR (Major Adverse Cardiac Events[Title/Abstract]) |
| #3 | ("Cardiovascular Diseases"[Mesh]) OR ((((((((((Cardiovascular Disease[Title/Abstract]) OR (Disease, Cardiovascular[Title/Abstract])) OR (Cardiac Events[Title/Abstract])) OR (Cardiac Event[Title/Abstract])) OR (Event, Cardiac[Title/Abstract])) OR (Adverse Cardiac Event[Title/Abstract])) OR (Adverse Cardiac Events[Title/Abstract])) OR (Cardiac Event, Adverse[Title/Abstract])) OR (Cardiac Events, Adverse[Title/Abstract])) OR (Major Adverse Cardiac Events[Title/Abstract])) |
| #4 | (((((((((((((((Artery Diseases, Coronary[Title/Abstract]) OR (Artery Disease, Coronary[Title/Abstract])) OR (Coronary Artery Diseases[Title/Abstract])) OR (Coronary Arteriosclerosis[Title/Abstract])) OR (Arterioscleroses, Coronary[Title/Abstract])) OR (Coronary Arterioscleroses[Title/Abstract])) OR (Arteriosclerosis, Coronary[Title/Abstract])) OR (Atherosclerosis, Coronary[Title/Abstract])) OR (Atheroscleroses, Coronary[Title/Abstract])) OR (Coronary Atheroscleroses[Title/Abstract])) OR (Coronary Atherosclerosis[Title/Abstract])) OR (Left Main Coronary Artery Disease[Title/Abstract])) OR (Left Main Coronary Disease[Title/Abstract])) OR (Left Main Disease[Title/Abstract])) OR (Left Main Diseases[Title/Abstract])) OR ("Coronary Artery Disease"[Mesh]) |
| #5 | (((((((((Coronary Diseases[Title/Abstract]) OR (Disease, Coronary[Title/Abstract])) OR (Diseases, Coronary[Title/Abstract])) OR (Coronary Heart Disease[Title/Abstract])) OR (Coronary Heart Diseases[Title/Abstract])) OR (Disease, Coronary Heart[Title/Abstract])) OR (Diseases, Coronary Heart[Title/Abstract])) OR (Heart Disease, Coronary[Title/Abstract])) OR (Heart Diseases, Coronary[Title/Abstract])) OR ("Coronary Disease"[Mesh]) |
| #6 | ("Stroke"[Mesh]) OR ((((((((((((((((((((((((((((Strokes[Title/Abstract]) OR (Cerebrovascular Accident[Title/Abstract])) OR (Cerebrovascular Accidents[Title/Abstract])) OR (Cerebral Stroke[Title/Abstract])) OR (Cerebral Strokes[Title/Abstract])) OR (Stroke, Cerebral[Title/Abstract])) OR (Strokes, Cerebral[Title/Abstract])) OR (Cerebrovascular Apoplexy[Title/Abstract])) OR (Apoplexy, Cerebrovascular[Title/Abstract])) OR (Vascular Accident, Brain[Title/Abstract])) OR (Brain Vascular Accident[Title/Abstract])) OR (Brain Vascular Accidents[Title/Abstract])) OR (Vascular Accidents, Brain[Title/Abstract])) OR (Cerebrovascular Stroke[Title/Abstract])) OR (Cerebrovascular Strokes[Title/Abstract])) OR (Stroke, Cerebrovascular[Title/Abstract])) OR (Strokes, Cerebrovascular[Title/Abstract])) OR (Apoplexy[Title/Abstract])) OR (CVA (Cerebrovascular Accident[Title/Abstract]))) OR (CVAs (Cerebrovascular Accident[Title/Abstract]))) OR (Stroke, Acute[Title/Abstract])) OR (Acute Stroke[Title/Abstract])) OR (Acute Strokes[Title/Abstract])) OR (Strokes, Acute[Title/Abstract])) OR (Cerebrovascular Accident, Acute[Title/Abstract])) OR (Acute Cerebrovascular Accident[Title/Abstract])) OR (Acute Cerebrovascular Accidents[Title/Abstract])) OR (Cerebrovascular Accidents, Acute[Title/Abstract])) |
| #7 | ("Ischemic Attack, Transient"[Mesh]) OR (((((((((((((((((((((((((((((((((((Brain TIA[Title/Abstract]) OR (TIA, Brain[Title/Abstract])) OR (TIA (Transient Ischemic Attack[Title/Abstract]))) OR (TIAs (Transient Ischemic Attack[Title/Abstract]))) OR (Transient Ischemic Attack[Title/Abstract])) OR (Attacks, Transient Ischemic[Title/Abstract])) OR (Attack, Transient Ischemic[Title/Abstract])) OR (Ischemic Attacks, Transient[Title/Abstract])) OR (Transient Ischemic Attacks[Title/Abstract])) OR (Cerebral Ischemia, Transient[Title/Abstract])) OR (Cerebral Ischemias, Transient[Title/Abstract])) OR (Ischemias, Transient Cerebral[Title/Abstract])) OR (Ischemia, Transient Cerebral[Title/Abstract])) OR (Transient Cerebral Ischemia[Title/Abstract])) OR (Transient Cerebral Ischemias[Title/Abstract])) OR (Brain Stem Ischemia, Transient[Title/Abstract])) OR (Transient Ischemic Attack, Brainstem[Title/Abstract])) OR (Transient Ischemic Attack, Brain Stem[Title/Abstract])) OR (Brain Stem Transient Ischemic Attack[Title/Abstract])) OR (Brainstem Ischemia, Transient[Title/Abstract])) OR (Brainstem Ischemias, Transient[Title/Abstract])) OR (Ischemias, Transient Brainstem[Title/Abstract])) OR (Ischemia, Transient Brainstem[Title/Abstract])) OR (Transient Brainstem Ischemia[Title/Abstract])) OR (Brainstem Transient Ischemic Attack[Title/Abstract])) OR (Crescendo Transient Ischemic Attacks[Title/Abstract])) OR (Transient Ischemic Attacks, Crescendo[Title/Abstract])) OR (Carotid Circulation Transient Ischemic Attack[Title/Abstract])) OR (Transient Ischemic Attack, Carotid Circulation[Title/Abstract])) OR (Posterior Circulation Transient Ischemic Attack[Title/Abstract])) OR (Transient Ischemic Attack, Posterior Circulation[Title/Abstract])) OR (Transient Ischemic Attack, Anterior Circulation[Title/Abstract])) OR (Anterior Circulation Transient Ischemic Attack[Title/Abstract])) OR (Transient Ischemic Attack, Vertebrobasilar Circulation[Title/Abstract])) OR (Vertebrobasilar Circulation Transient Ischemic Attack[Title/Abstract])) |
| #8 | ("Peripheral Arterial Disease"[Mesh]) OR (((((((((((Arterial Disease, Peripheral[Title/Abstract]) OR (Arterial Diseases, Peripheral[Title/Abstract])) OR (Disease, Peripheral Arterial[Title/Abstract])) OR (Diseases, Peripheral Arterial[Title/Abstract])) OR (Peripheral Arterial Diseases[Title/Abstract])) OR (Peripheral Artery Disease[Title/Abstract])) OR (Artery Disease, Peripheral[Title/Abstract])) OR (Artery Diseases, Peripheral[Title/Abstract])) OR (Disease, Peripheral Artery[Title/Abstract])) OR (Diseases, Peripheral Artery[Title/Abstract])) OR (Peripheral Artery Diseases[Title/Abstract])) |
| #9 | (CVD[Title/Abstract]))OR (CAD[Title/Abstract])) OR (CHD[Title/Abstract]) |
| #10 | #3 OR #4 OR #5 OR #6 OR #7 OR #8 OR #9 |
| #11 | ("METS-IR"[Title/Abstract]) OR ("metabolic score for insulin resistance"[Title/Abstract]) |
| #12 | #10 AND #11 |
| **Embase** | |
| #1 | cardiovascular disease'/exp |
| #2 | cardiovascular disease':ab,ti OR 'disease,cardiovascular':ab,ti OR 'cardiac events':ab,ti OR 'cardiac event':ab,ti OR 'event,cardiac':ab,ti OR 'adverse cardiac event':ab,ti OR 'adverse cardiac events':ab,ti OR 'cardiac event, adverse':ab,ti OR 'cardiac events,adverse':ab,ti OR 'major adverse cardiac events':ab,ti |
| #3 | coronary artery disease'/exp |
| #4 | artery diseases, coronary':ab,ti OR 'artery disease, coronary':ab,ti OR 'coronary artery diseases':ab,ti OR 'coronary arteriosclerosis':ab,ti OR 'arterioscleroses,coronary':ab,ti OR 'coronary arterioscleroses':ab,ti OR 'arteriosclerosis,coronary':ab,ti OR 'atherosclerosis,coronary':ab,ti OR 'atheroscleroses,coronary':ab,ti OR 'coronary atheroscleroses':ab,ti OR 'coronary atherosclerosis':ab,ti OR 'left main coronary artery disease':ab,ti OR 'left main coronary disease':ab,ti OR 'left main disease':ab,ti OR 'left main diseases':ab,ti |
| #5 | coronary diseases':ab,ti OR 'disease,coronary':ab,ti OR 'diseases, coronary':ab,ti OR 'coronary heart disease':ab,ti OR 'coronary heart diseases':ab,ti OR 'disease, coronary heart':ab,ti OR 'diseases, coronary heart':ab,ti OR 'heart disease, coronary':ab,ti OR 'heart diseases, coronary':ab,ti |
| #6 | cerebrovascular accident'/exp |
| #7 | strokes':ab,ti OR 'stroke':ab,ti OR 'cerebrovascular accidents':ab,ti OR 'cerebral stroke':ab,ti OR 'cerebral strokes':ab,ti OR 'stroke, cerebral':ab,ti OR 'strokes,cerebral':ab,ti OR 'cerebrovascular apoplexy':ab,ti OR 'apoplexy,cerebrovascular':ab,ti OR 'vascular accident,brain':ab,ti OR 'brain vascular accident':ab,ti OR 'brain vascular accidents':ab,ti OR 'vascular accidents, brain':ab,ti OR 'cerebrovascular stroke':ab,ti OR 'cerebrovascular strokes':ab,ti OR 'stroke, cerebrovascular':ab,ti OR 'strokes,cerebrovascular':ab,ti OR 'apoplexy':ab,ti OR 'cva (cerebrovascular accident':ab,ti OR 'cvas(cerebrovascular accident':ab,ti OR 'stroke,acute':ab,ti OR 'acute stroke':ab,ti OR 'acute strokes':ab,ti OR 'strokes, acute':ab,ti OR 'cerebrovascular accident, acute':ab,ti OR 'acute cerebrovascular accident':ab,ti OR 'acute cerebrovascular accidents':ab,ti OR 'cerebrovascular accidents, acute' |
| #8 | transient ischemic attack'/exp |
| #9 | brain tia':ab,ti OR 'tia, brain':ab,ti OR 'tia(transient ischemic attack)':ab,ti OR 'tias(transient ischemic attack)':ab,ti OR 'ischemic attack, transient':ab,ti OR 'attacks, transient ischemic':ab,ti OR 'attack, transient ischemic':ab,ti OR 'ischemic attacks,transient':ab,ti OR 'transient ischemic attacks':ab,ti OR 'cerebral ischemia,transient':ab,ti OR 'cerebral ischemias,transient':ab,ti OR 'ischemias, transient cerebral':ab,ti OR 'ischemia, transient cerebral':ab,ti OR 'transient cerebral ischemia':ab,ti OR 'transient cerebral ischemias':ab,ti OR 'brain stem ischemia,transient':ab,ti OR 'transient ischemic attack,brainstem':ab,ti OR 'transient ischemic attack,brain stem':ab,ti OR 'brain stem transient ischemic attack':ab,ti OR 'brainstem ischemia,transient':ab,ti OR 'brainstem ischemias,transient':ab,ti OR 'ischemias, transient brainstem':ab,ti OR 'ischemia, transient brainstem':ab,ti OR 'transient brainstem ischemia':ab,ti OR 'brainstem transient ischemic attack':ab,ti OR 'crescendo transient ischemic attacks':ab,ti OR 'transient ischemic attacks,crescendo':ab,ti OR 'carotid circulation transient ischemic attack':ab,ti OR 'transient ischemic attack, carotid circulation':ab,ti OR 'posterior circulation transient ischemic attack':ab,ti OR 'transient ischemic attack,posterior circulation':ab,ti OR 'transient ischemic attack, anterior circulation':ab,ti OR 'anterior circulation transient ischemic attack':ab,ti OR 'transient ischemic attack,vertebrobasilar circulation':ab,ti OR 'vertebrobasilar circulation transient ischemic attack':ab,ti |
| #10 | peripheral arterial disease'/exp |
| #11 | arterial disease, peripheral':ab,ti OR 'arterial diseases, peripheral':ab,ti OR 'disease,peripheral arterial':ab,ti OR 'diseases,peripheral arterial':ab,ti OR 'peripheral arterial diseases':ab,ti OR 'peripheral artery disease':ab,ti OR 'artery disease,peripheral':ab,ti OR 'artery diseases,peripheral':ab,ti OR 'disease, peripheral artery':ab,ti OR 'diseases, peripheral artery':ab,ti OR 'peripheral artery diseases':ab,ti |
| #12 | cvd':ab,ti OR 'cad':ab,ti OR 'chd':ab,ti |
| #13 | #1 OR #2 OR #3 OR #4 OR #5 OR #6 OR #7 OR #8 OR #9 OR #10 OR #11 OR #12 |
| #14 | mets-ir':ab,ti OR 'metabolic score for insulin resistance':ab,ti |
| #15 | #13 AND #14 |
| **Cochrane** | |
| #1 | MeSH descriptor: [Cardiovascular Diseases] explode all trees |
| #2 | (Cardiovascular Disease):ti,ab,kw OR (Disease, Cardiovascular):ti,ab,kw OR (Cardiac Events):ti,ab,kw OR (Cardiac Event):ti,ab,kw OR (Event, Cardiac):ti,ab,kw OR (Adverse Cardiac Event):ti,ab,kw OR (Adverse Cardiac Events):ti,ab,kw OR (Cardiac Event, Adverse):ti,ab,kw OR (Cardiac Events, Adverse):ti,ab,kw OR (Major Adverse Cardiac Events):ti,ab,kw |
| #3 | MeSH descriptor: [Coronary Artery Disease] explode all trees |
| #4 | (Artery Diseases, Coronary):ti,ab,kw OR (Artery Disease, Coronary):ti,ab,kw OR (Coronary Artery Diseases):ti,ab,kw OR (Coronary Arteriosclerosis):ti,ab,kw OR (Arterioscleroses, Coronary):ti,ab,kw OR (Coronary Arterioscleroses):ti,ab,kw OR (Arteriosclerosis, Coronary):ti,ab,kw OR (Atherosclerosis, Coronary):ti,ab,kw OR (Atheroscleroses, Coronary):ti,ab,kw OR (Coronary Atheroscleroses):ti,ab,kw OR (Coronary Atherosclerosis):ti,ab,kw OR (Left Main Coronary Artery Disease):ti,ab,kw OR (Left Main Coronary Disease):ti,ab,kw OR (Left Main Disease):ti,ab,kw OR (Left Main Diseases):ti,ab,kw |
| #5 | MeSH descriptor: [Coronary Disease] explode all trees |
| #6 | (Coronary Diseases):ti,ab,kw OR (Disease, Coronary):ti,ab,kw OR (Diseases, Coronary):ti,ab,kw OR (Coronary Heart Disease):ti,ab,kw OR (Coronary Heart Diseases):ti,ab,kw OR (Disease, Coronary Heart):ti,ab,kw OR (Diseases, Coronary Heart):ti,ab,kw OR (Heart Disease, Coronary):ti,ab,kw OR (Heart Diseases, Coronary):ti,ab,kw |
| #7 | MeSH descriptor: [Stroke] explode all trees |
| #8 | (Strokes):ti,ab,kw OR (Cerebrovascular accident):ti,ab,kw OR (Cerebrovascular Accidents):ti,ab,kw OR (Cerebral Stroke):ti,ab,kw OR (Cerebral Strokes):ti,ab,kw OR (Stroke, Cerebral):ti,ab,kw OR (Strokes, Cerebral):ti,ab,kw OR (Cerebrovascular Apoplexy):ti,ab,kw OR (Apoplexy, Cerebrovascular):ti,ab,kw OR (Vascular Accident, Brain):ti,ab,kw OR (Brain Vascular Accident):ti,ab,kw OR (Brain Vascular Accidents):ti,ab,kw OR (Vascular Accidents, Brain):ti,ab,kw OR (Cerebrovascular Stroke):ti,ab,kw OR (Cerebrovascular Strokes):ti,ab,kw OR (Stroke, Cerebrovascular):ti,ab,kw OR (Strokes, Cerebrovascular):ti,ab,kw OR (Apoplexy):ti,ab,kw OR (CVA):ti,ab,kw OR (CVAs):ti,ab,kw OR (Stroke, Acute):ti,ab,kw OR (Acute Stroke):ti,ab,kw OR (Acute Strokes):ti,ab,kw OR (Strokes, Acute):ti,ab,kw OR (Cerebrovascular Accident, Acute):ti,ab,kw OR (Acute Cerebrovascular Accident):ti,ab,kw OR (Acute Cerebrovascular Accidents):ti,ab,kw OR (Cerebrovascular Accidents, Acute):ti,ab,kw |
| #9 | MeSH descriptor: [Ischemic Attack, Transient] explode all trees |
| #10 | (Brain TIA):ti,ab,kw OR (TIA, Brain):ti,ab,kw OR (TIA (Transient Ischemic Attack)):ti,ab,kw OR (TIAs (Transient Ischemic Attack)):ti,ab,kw OR (Transient Ischemic Attack):ti,ab,kw OR (Attacks, Transient Ischemic):ti,ab,kw OR (Attack, Transient Ischemic):ti,ab,kw OR (Ischemic Attacks, Transient):ti,ab,kw OR (Transient Ischemic Attacks):ti,ab,kw OR (Cerebral Ischemia, Transient):ti,ab,kw OR (Cerebral Ischemias, Transient):ti,ab,kw OR (Ischemias, Transient Cerebral):ti,ab,kw OR (Ischemia, Transient Cerebral):ti,ab,kw OR (Transient Cerebral Ischemia):ti,ab,kw OR (Transient Cerebral Ischemias):ti,ab,kw OR (Brain Stem Ischemia, Transient):ti,ab,kw OR (Transient Ischemic Attack, Brainstem):ti,ab,kw OR (Transient Ischemic Attack, Brain Stem):ti,ab,kw OR (Brain Stem Transient Ischemic Attack):ti,ab,kw OR (Brainstem Ischemia, Transient):ti,ab,kw OR (Brainstem Ischemias, Transient):ti,ab,kw OR (Ischemias, Transient Brainstem):ti,ab,kw OR (Ischemia, Transient Brainstem):ti,ab,kw OR (Transient Brainstem Ischemia):ti,ab,kw OR (Brainstem Transient Ischemic Attack):ti,ab,kw OR (Crescendo Transient Ischemic Attacks):ti,ab,kw OR (Transient Ischemic Attacks, Crescendo):ti,ab,kw OR (Carotid Circulation Transient Ischemic Attack):ti,ab,kw OR (Transient Ischemic Attack, Carotid Circulation):ti,ab,kw OR (Posterior Circulation Transient Ischemic Attack):ti,ab,kw OR (Transient Ischemic Attack, Posterior Circulation):ti,ab,kw OR (Transient Ischemic Attack, Anterior Circulation):ti,ab,kw OR (Anterior Circulation Transient Ischemic Attack):ti,ab,kw OR (Transient Ischemic Attack, Vertebrobasilar Circulation):ti,ab,kw OR (Vertebrobasilar Circulation Transient Ischemic Attack):ti,ab,kw |
| #11 | MeSH descriptor: [Peripheral Arterial Disease] explode all trees |
| #12 | (Arterial Disease, Peripheral):ti,ab,kw OR (Arterial Diseases, Peripheral):ti,ab,kw OR (Disease, Peripheral Arterial):ti,ab,kw OR (Diseases, Peripheral Arterial):ti,ab,kw OR (Peripheral Arterial Diseases):ti,ab,kw OR (Peripheral Artery Disease):ti,ab,kw OR (Artery Disease, Peripheral):ti,ab,kw OR (Artery Diseases, Peripheral):ti,ab,kw OR (Disease, Peripheral Artery):ti,ab,kw OR (Diseases, Peripheral Artery):ti,ab,kw OR (Peripheral Artery Diseases):ti,ab,kw |
| #13 | (CVD):ti,ab,kw OR (CAD):ti,ab,kw OR (CHD):ti,ab,kw |
| #14 | #1 OR #2 OR #3 OR #4 OR #5 OR #6 OR #7 OR #8 OR #9 OR #10 OR #11 OR #12 OR #13 |
| #15 | (METS-IR):ti,ab,kw OR (metabolic score for insulin resistance):ti,ab,kw |
| #16 | #14 AND #15 |
| **Web of Science** | |
| #1 | TS=(Cardiovascular Diseases OR Cardiovascular Disease OR Disease, Cardiovascular OR Cardiac Events OR Cardiac Event OR Event, Cardiac OR Adverse Cardiac Event OR Adverse Cardiac Events OR Cardiac Event, Adverse OR Cardiac Events, Adverse OR Major Adverse Cardiac Events) and Preprint Citation Index (Exclude – Database) |
| #2 | TS=(Artery Diseases, Coronary OR Artery Disease, Coronary OR Coronary Artery Diseases OR Coronary Arteriosclerosis OR Arterioscleroses, Coronary OR Coronary Arterioscleroses OR Arteriosclerosis, Coronary OR Atherosclerosis, Coronary OR Atheroscleroses, Coronary OR Coronary Atheroscleroses OR Coronary Atherosclerosis OR Left Main Coronary Artery Disease OR Left Main Coronary Disease OR Left Main Disease OR Left Main Diseases OR Coronary Artery Disease) and Preprint Citation Index (Exclude – Database) |
| #3 | TS=(Coronary Diseases OR Disease, Coronary OR Diseases, Coronary OR Coronary Heart Disease OR Coronary Heart Diseases OR Disease, Coronary Heart OR Diseases, Coronary Heart OR Heart Disease, Coronary OR Heart Diseases, Coronary OR Coronary Disease) and Preprint Citation Index (Exclude – Database) |
| #4 | TS=(Strokes OR Cerebrovascular Accident OR Cerebrovascular Accidents OR Cerebral Stroke OR Cerebral Strokes OR Stroke, Cerebral OR Strokes, Cerebral OR Cerebrovascular Apoplexy OR Apoplexy, Cerebrovascular OR Vascular Accident, Brain OR Brain Vascular Accident OR Brain Vascular Accidents OR Vascular Accidents, Brain OR Cerebrovascular Stroke OR Cerebrovascular Strokes OR Stroke, Cerebrovascular OR Strokes, Cerebrovascular OR Apoplexy OR CVA (Cerebrovascular Accident) OR CVAs (Cerebrovascular Accident) OR Stroke, Acute OR Acute Stroke OR Acute Strokes OR Strokes, Acute OR Cerebrovascular Accident, Acute OR Acute Cerebrovascular Accident OR Acute Cerebrovascular Accidents OR Cerebrovascular Accidents, Acute OR Stroke) and Preprint Citation Index (Exclude – Database) |
| #5 | TS=(Ischemic Attack, Transient OR Brain TIA OR TIA, Brain OR TIA (Transient Ischemic Attack) OR TIAs (Transient Ischemic Attack) OR Ischemic Attack, Transient OR Attacks, Transient Ischemic OR Attack, Transient Ischemic OR Ischemic Attacks, Transient OR Transient Ischemic Attacks OR Cerebral Ischemia, Transient OR Cerebral Ischemias, Transient OR Ischemias, Transient Cerebral OR Ischemia, Transient Cerebral OR Transient Cerebral Ischemia OR Transient Cerebral Ischemias OR Brain Stem Ischemia, Transient OR Transient Ischemic Attack, Brainstem OR Transient Ischemic Attack, Brain Stem OR Brain Stem Transient Ischemic Attack OR Brainstem Ischemia, Transient OR Brainstem Ischemias, Transient OR Ischemias, Transient Brainstem OR Ischemia, Transient Brainstem OR Transient Brainstem Ischemia OR Brainstem Transient Ischemic Attack OR Crescendo Transient Ischemic Attacks OR Transient Ischemic Attacks, Crescendo OR Carotid Circulation Transient Ischemic Attack OR Transient Ischemic Attack, Carotid Circulation OR Posterior Circulation Transient Ischemic Attack OR Transient Ischemic Attack, Posterior Circulation OR Transient Ischemic Attack, Anterior Circulation OR Anterior Circulation Transient Ischemic Attack OR Transient Ischemic Attack, Vertebrobasilar Circulation OR Vertebrobasilar Circulation Transient Ischemic Attack) and Preprint Citation Index (Exclude – Database) |
| #6 | TS=(Peripheral Arterial Disease OR Arterial Disease, Peripheral OR Arterial Diseases, Peripheral OR Disease, Peripheral Arterial OR Diseases, Peripheral Arterial OR Peripheral Arterial Diseases OR Peripheral Artery Disease OR Artery Disease, Peripheral OR Artery Diseases, Peripheral OR Disease, Peripheral Artery OR Diseases, Peripheral Artery OR Peripheral Artery Diseases) and Preprint Citation Index (Exclude – Database) |
| #7 | TS=(CAD OR CHD OR CVD) and Preprint Citation Index (Exclude – Database) |
| #8 | #7 OR #6 OR #5 OR #4 OR #3 OR #2 OR #1 and Preprint Citation Index (Exclude – Database) |
| #9 | TS=(“METS-IR” OR “metabolic score for insulin resistance”) and Preprint Citation Index (Exclude – Database) |
| #10 | #9 AND #8 and Preprint Citation Index (Exclude – Database) |

**Fig. S1** Trim-and-fill analysis results for the association between METS-IR and composite CVD risk, analyzed as a categorical variable (A) and continuous variable (B).


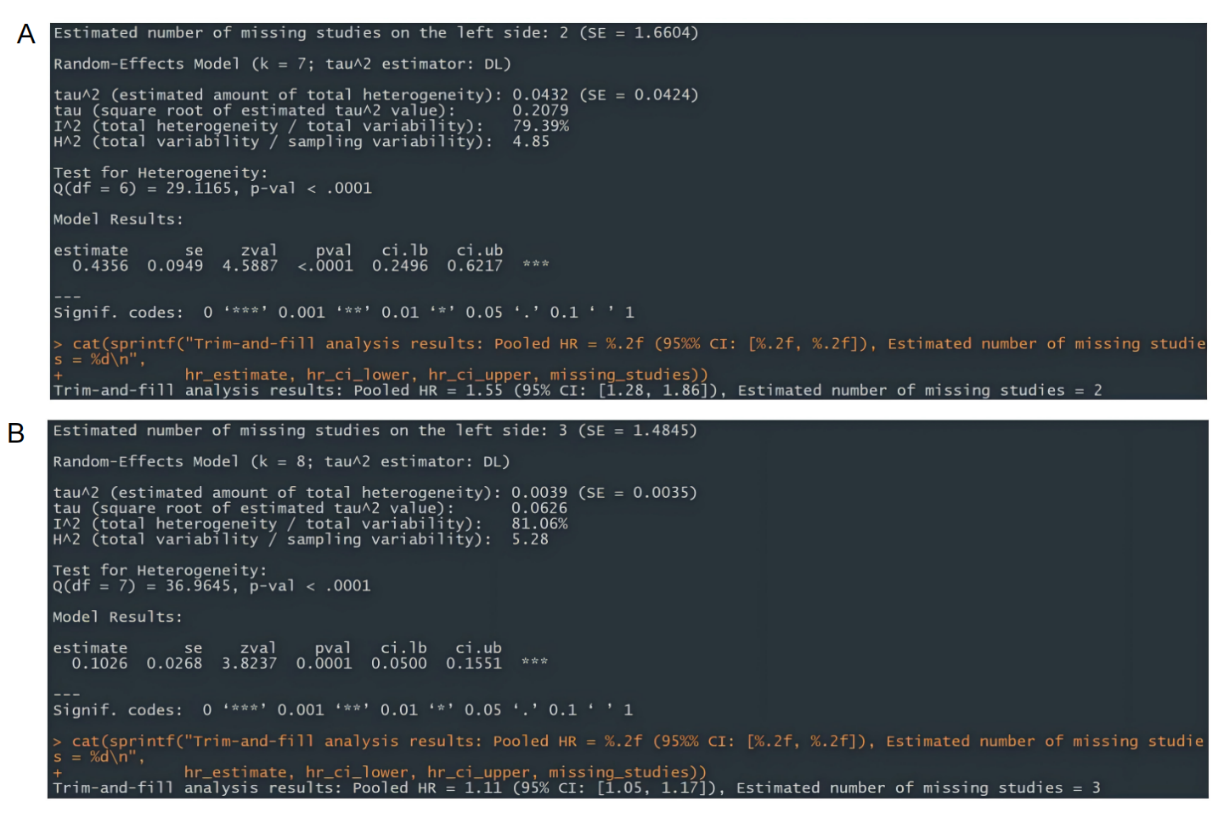


Abbreviation: METS-IR, metabolic score for insulin resistance; CVD, cardiovascular disease.

**Fig. S2** Trim-and-fill analysis results for the association between METS-IR and CAD risk, analyzed as a categorical variable (A) and continuous variable (B).


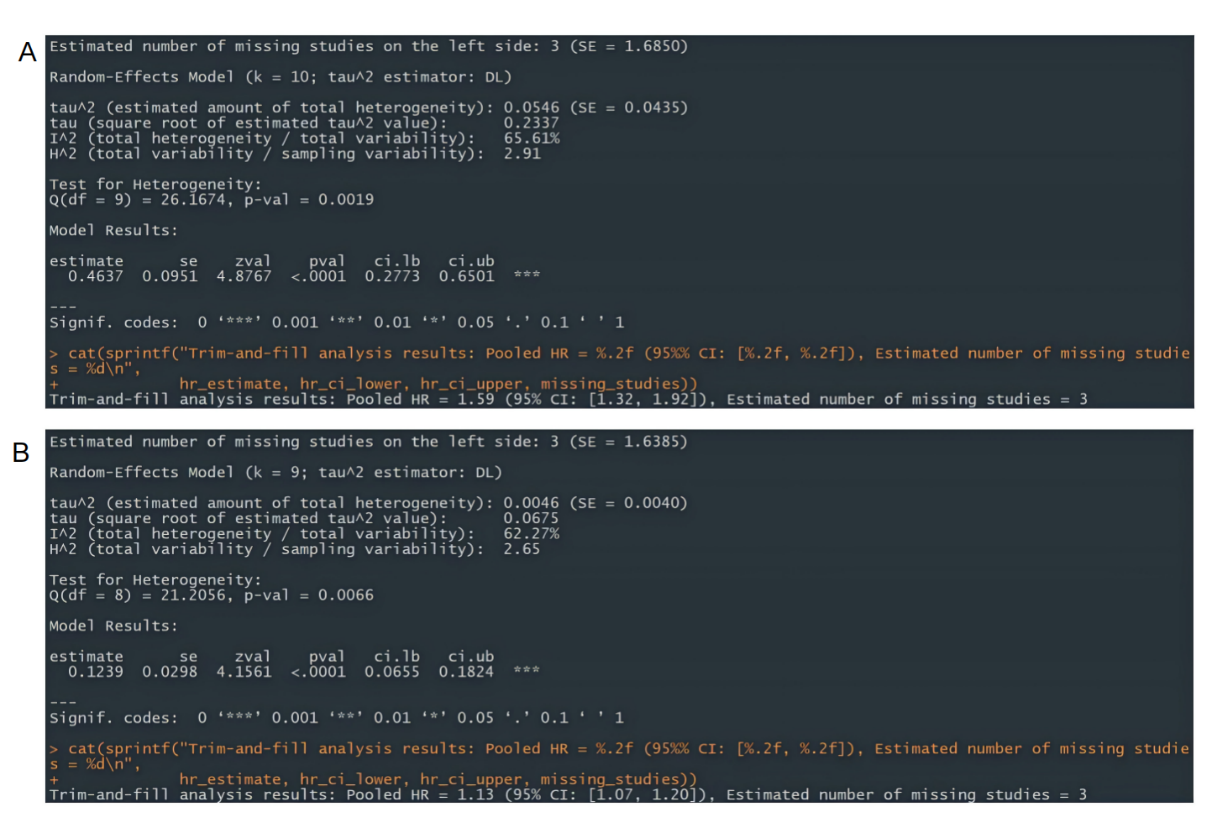


Abbreviation: METS-IR, metabolic score for insulin resistance; CAD, coronary artery disease.

**Fig. S3** Trim-and-fill analysis results for the association between METS-IR and stroke risk, analyzed as a categorical variable (A) and continuous variable (B).


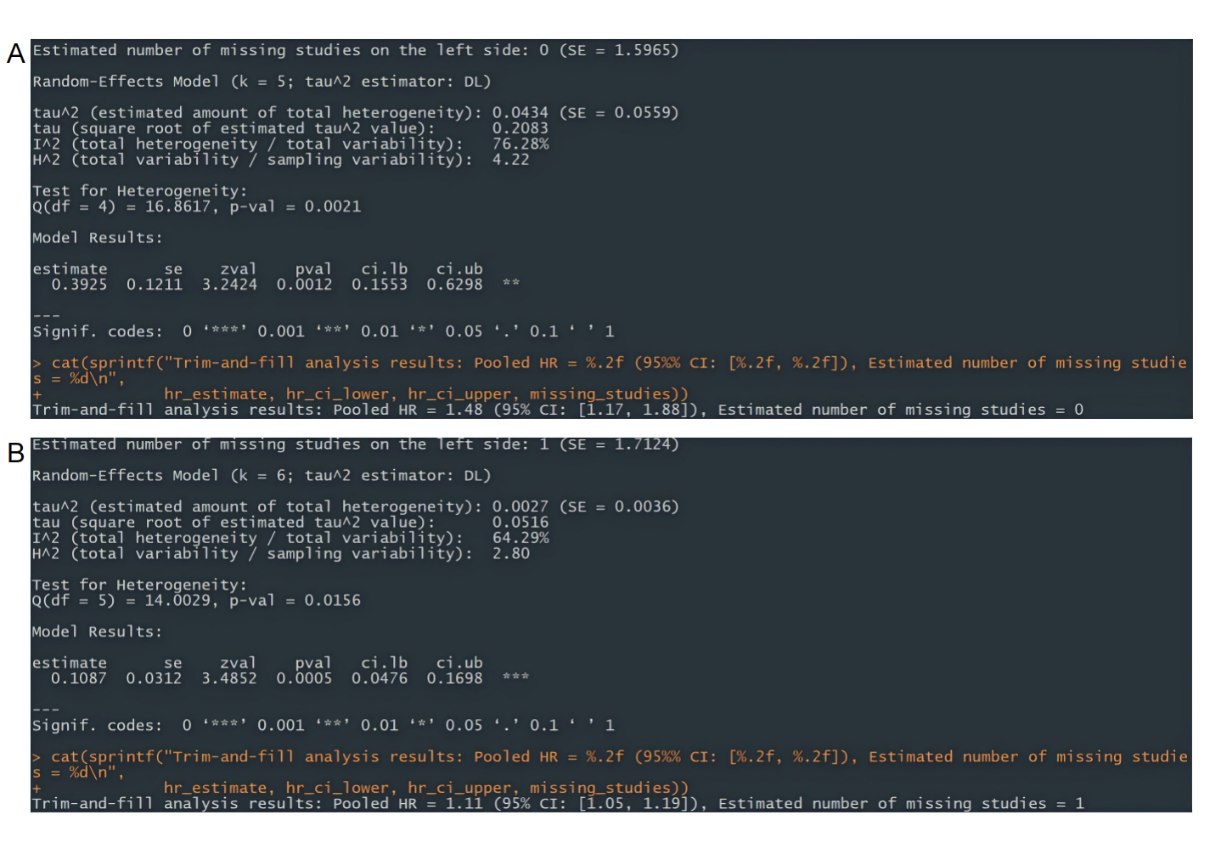


Abbreviation: METS-IR, metabolic score for insulin resistance.
